# Inferring Epidemic Contact Structure from Phylogenetic Trees

**DOI:** 10.1371/journal.pcbi.1002413

**Published:** 2012-03-08

**Authors:** Gabriel E. Leventhal, Roger Kouyos, Tanja Stadler, Viktor von Wyl, Sabine Yerly, Jürg Böni, Cristina Cellerai, Thomas Klimkait, Huldrych F. Günthard, Sebastian Bonhoeffer

**Affiliations:** 1 Institute of Integrative Biology, ETH Zurich, Zurich, Switzerland; 2 Department of Ecology and Evolutionary Biology, Princeton University, Princeton, New Jersey, United States of America; 3 Division of Infectious Diseases and Hospital Epidemiology, University Hospital Zurich, Zurich, Switzerland; 4 Laboratory of Virology and AIDS Center, Geneva University Hospital, Geneva, Switzerland; 5 Swiss National Center for Retroviruses, Institute of Medical Virology, University of Zurich, Zurich, Switzerland; 6 Service of Immunology and Allergy, Lausanne University Hospital, Lausanne, Switzerland; 7 Institute of Medical Microbiology, Department Biomedicine, University of Basel, Basel, Switzerland; University of New South Wales, Australia

## Abstract

Contact structure is believed to have a large impact on epidemic spreading and consequently using networks to model such contact structure continues to gain interest in epidemiology. However, detailed knowledge of the exact contact structure underlying real epidemics is limited. Here we address the question whether the structure of the contact network leaves a detectable genetic fingerprint in the pathogen population. To this end we compare phylogenies generated by disease outbreaks in simulated populations with different types of contact networks. We find that the shape of these phylogenies strongly depends on contact structure. In particular, measures of tree imbalance allow us to quantify to what extent the contact structure underlying an epidemic deviates from a null model contact network and illustrate this in the case of random mixing. Using a phylogeny from the Swiss HIV epidemic, we show that this epidemic has a significantly more unbalanced tree than would be expected from random mixing.

## Author Summary

One of the recent key innovations in the epidemiology of infectious diseases was the incorporation of explicit contact structure (i.e. who can infect whom) into epidemiological models. Theoretical studies have generated a broad consensus in the field that knowledge of the contact network may help to greatly improve the control of the spread of epidemics. The key problem in the field, however, is that we lack knowledge regarding the actual contact structure underlying real epidemics. Much research is focused on trying to reconstruct actual contact networks in various ways (mobile phone usage data, electronic devices that measure physical proximity, patient interviews, etc). All of these approaches are highly labour intensive and are fraught with many difficulties. Here, we present a new approach which is based on readily available sequence data. Using the Swiss HIV epidemic as an example, we show that it displays strong indications of a underlying contact structure that strongly differs from random interactions, thus undercutting the assumption of random mixing which is commonly made in epidemiological models.

## Introduction

Infectious disease epidemiology has a longstanding history of mathematical modeling. Simple population dynamical models assuming random-mixing between infected and susceptible individuals have yielded important insights into the dynamics and control of infectious diseases [1]. The assumption of random mixing has been made primarily for reasons of mathematical tractability, but it is unclear under which conditions this assumption is actually justified.

To account for the effects of non-random mixing networks that describe the explicit contact structure are increasingly being incorporated into models of infectious disease dynamics [2]–[7]. These models showed that important epidemiological quantities such as the size of an epidemic or requirements for an epidemic to spread depend sensitively on the contact structure [8]–[11]. While this has led to important conceptual insights regarding disease dynamics and control, the applicability of these results to real world situations has been hampered by the paucity of data on actual contact networks. Great efforts are currently underway to infer contact structure from various sources of data [12]–[16]. However, most approaches for the reconstruction of contact networks are highly labor-intensive. These approaches are also all based on host contact structure which is not always easily translated into contacts that are relevant for transmission. While some studies have focused on differentiating contacts that may or may not result in transmission [15], there are many factors that can cause discrepencies between interactions of hosts and interactations that are relevant for transmission.

Considerable efforts have recently been made to link methods of phylogenetic analysis with epidemiological models resulting in a new research area termed phylodynamics [17]–[19]. If the evolutionary rate of a pathogen is sufficiently fast, then it is conceivable that also the contact structure underlying an epidemic leaves a traceable fingerprint in the genetic structure of the pathogen population [20], [21]. This contact network will only contain those contacts that are relevant for disease transmission and therefore could circuimvent problems of translating host contacts into disease contacts.

Using simulations of pathogen populations spreading on contact networks generated by various network models we compare the resulting phylogenetic trees. We find that quantitative measurements of tree shape such as the Sackin index contain information that can be used to differentiate between different classes of contact structures. Such descriptive measures of tree topology have previously been successfully used to infer evolutionary processes from phylogenetic trees [22]–. Most importantly, the Sackin index can be used to test whether the contact structure significantly deviates from what would be expected under random mixing.

## Results

### Network structure and tree balance

To illustrate the effect of contact structure on the resulting phylogenetic tree, we perform simulations of epidemic outbreaks for three different network models: (a) the Erdös-Rényi (ER) random graph [29], (b) the Barabási-Albert (BA) graph [30] and (c) the Watts-Strogatz (WS) graph [31] with a low rewiring probability, 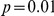 (see Methods). Both the BA and the WS are representative for two important aspects of contact heterogeneity. Networks generated by the BA model have both a large variance in the degree distribution as well as short mean path lengths. Networks generated by the WS model have low degree distribution variance and long mean path lengths. We compare these network models to a full graph (FG), which corresponds to a model with random mixing.

Each of the three network models can be tuned with different parameters. The ER model generates networks with Poisson degree distributions where the mean can be varied. The BA model produces scale-free networks with a power-law degree distribution. The WS model produces networks that have a high degree of local clustering, but a degree distribution that lies between a Dirac distribution (all nodes have the same degree) and a Poisson distribution.

**Figure 1:**
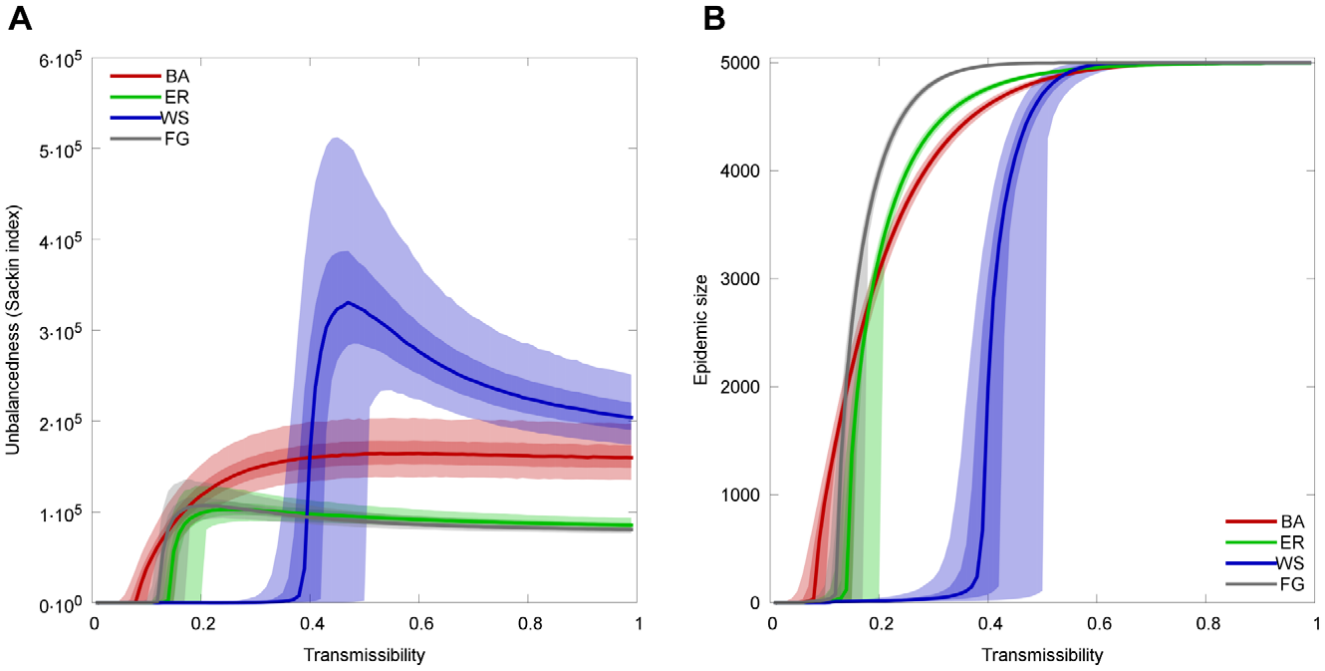
Imbalance of the infection tree for contact networks generated by three different models. Panel A shows the Sackin index as a measure of tree imbalance. Panel B shows the size of the epidemic outbreak for each of the network models at different values of transmissibility 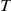. All three models have the same mean number of neighbors 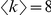. For the BA model, each vertex added in the preferential attachment is connected to 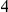 nodes in the existing network, resulting in a mean degree of 8. The WS networks start with a ring lattice where each node is connected to its 4 closest neighbors on each side. Every link is then rewired with probability 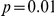. The light shaded area show the values lying between the 2.5-th and the 97.5-th percentile, the dark shaded area the values between the 25th and the 75th percentile and the solid lines are the mean of the simulations. Each data point corresponds to 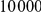 simulation runs on independent graphs. A plot showing the normalized Sackin index for these three network models can be found in the supporting text S2.

We track the exact spreading pattern (i.e. who infects whom) of a susceptible-infected-removed (SIR) epidemic for 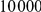 different networks generated by each model to obtain the infection tree for each of these networks. The parameters for the network models are chosen such that all networks have the same mean degree, 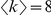, yet different degree distributions, path length distributions and clustering coefficients. Figure 1A shows the imbalance measured by the Sackin index of the resulting infection trees for the three network models and the full graph at different values of 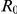, captured by the transmissibility 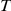 (see Methods for detailed definitions of the Sackin index and the transmissibility).

For large values of 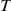 (large 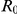) the whole network is infected, independent of the contact structure. Not surprisingly, the epidemic size is similar for all network types in this parameter range, since almost all individuals in the population eventually become infected before the epidemic dies out. The balancedness of the resulting trees, however, differ significantly for the three networks types. The ER model is virtually indistinguishable from the random mixing model (FG). For sufficiently large 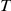 the BA model has higher imbalance than the ER and FG. Finally, the most striking difference in imbalance is observed for the WS.

For low 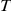 (low 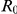) the imbalance vanishes for all networks for the simple reason that no epidemic outbreak occurs (see Figure 1B). Interestingly, the imbalance is generally largest at 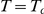, where the transmissibility is just large enough for an epidemic outbreak to occur. In this case each individual infects just one other individual on average, which results in an infection tree that continuously mostly branches off to one side and thus is maximally unbalanced (Figure S2).

For all network types except the BA model, the imbalance of the transmission tree is maximal for values of 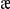 right around 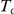, but then converges to a smaller value as 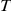 approaches unity. This can be explained by the fact that the SIR infection process is equivalent to a birth-death process. When 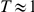, the death rate is de facto zero (birth rate 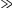 death rate) and thus all lineages survive to the end. If the death rate vanishes the expected imbalance of the resulting transmission trees is minimal and is given by the Yule model (see Methods).

The level of imbalance of the transmission trees for the different network types shown in Figure 1 obviously depends on the choice of the network model parameters. In the following we will investigate how imbalance depends on the average number of neighbors and on local connectivity. Moreover, we henceforth use the expected Sackin index given by the Yule model to define a normalized Sackin index (see Methods), which has an expected value of zero for infection trees based on an SIR model with death rate zero.

### The effect of the mean number of neighbors on tree imbalance

**Figure 2:**
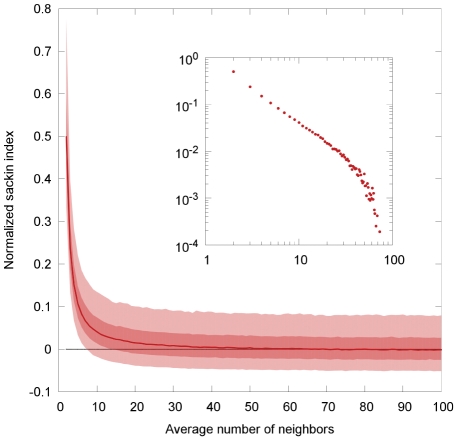
Imbalance (normalized Sackin index) of the infection tree for ER random graphs with varying number of average neighbors. The light shaded area show the values lying between the 2.5-th and 97.5-th percentile, the dark shaded area those between the 25th and 75th percentile. The dashed line is the expected value of the imbalance for a tree with the same number of leaves under the Yule model (equation (7)). The transmissibility is chosen 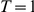. The inset show the same data points on a log-log scale.

We focus on the ER graph because in the limit of a large number of neighbors this model is expected to converge to the random mixing model. Furthermore, to eliminate contributions to imbalance resulting from a non-zero death rate we show the results for 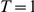. Figure 2 shows the imbalance for an ER model with 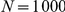 nodes and an average number of neighbors 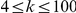. The effect of 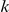 on networks generated by the WS and BA model are reported in the supporting text S2.

Increasing the mean number of neighbors essentially increases the number of infections caused by a single individual and therefore the imbalance is expected to decreases with increasing number of neighbors. This is confirmed by the results presented in Figure 2. A small average number of neighbors results in more unbalanced transmission trees for a reason that is similar to why nonzero death rates increase imbalance. Once a node has infected all of its 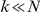 neighbors, it can no longer infect anyone else and is essentially removed from the system despite remaining infectious.

### The effect of mean path length on tree imbalance

**Figure 3:**
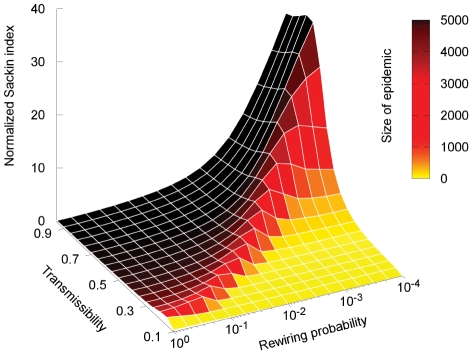
Normalized Sackin index for epidemics occurring on Watts-Strogatz graphs with varying rewiring probability. The total size of the population is 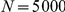 and the color shows the size of the epidemic outbreak. The network is constructed by connected each node to its 8 closest neighbors on a ring lattice, and then rewiring each link with a probability 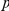. At low rewiring and transmission probabilities, 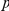 and 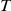 respectively, the epidemic only infects a small portion of the population and thus the Sackin index remains fairly small. Imbalance is largest for values of 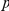 close to the critical value 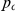 where the epidemic transition occurs.

In the WS model, the mean path length is directly related to the rewiring probability [31]. The WS model with rewiring probability 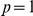 essentially generates the same type of network as the ER model. Therefore the imbalance of the transmission trees resulting from epidemics spreading on such networks should converge with increasing rewiring probability to the same value as for ER random graphs. Figure 3 shows imbalance as a function of the rewiring probability and transmissibility.

We identify two limiting cases for the imbalance of the epidemic. For values of 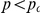 there is essentially no epidemic outbreak and the imbalance remains small. For values of 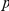 close to but larger than 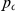 an epidemic can occur and the imbalance is maximal. As 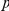 increases further, the number of shortcuts in the network increases and the mean path length decreases, as does the imbalance. For values substantially larger than 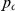 the network converges to something similar to an ER graph and the hence normalized imbalance converges to zero (for 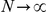 and 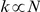) or to a fixed value for finite populations and small mean degree.

### Analytical insight

In the supporting text S1 we derive an analytical approximation for the normalized Sackin index given the transmission network (see Figure S1),
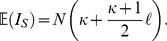
(1)Here, 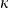 is the average number of infections caused by an infected individual until that individual is removed (i.e. the excess degree in the transmission network) and 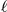 is the mean shortest path length in the transmission network. This equation shows that assuming the transmission network were known, imbalance depends on one hand on the mean path length, 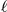, and on the other hand on the average excess degree 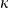. For networks generated by the configuration model, 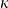 depends on the first and second moment of the degree distribution. BA networks are characterized by a large degree distribution variance, as well as a short mean path length. For low rewiring probabilities, WS networks have small degree distribution variances and large mean path lengths. These observations together with the analytical approximation in equation (1) can help explain why it is not always possible to distinguish between the BA and WS models when considering the Sackin index as a measure of tree topology (see Figure 1). This ambiguity is most pronounced when considering two idealized networks: a chain and a star. These two topologically very different networks would result in identical transmission trees (see Figure S2) and therefore be indistinguishable using tree imbalance alone.

Note that 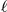 and 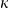 in equation (1) refer to the transmission network rather than the actual contact network. The connection between contact networks and transmission networks has recently been studied in the context of epidemic percolation networks [32]. Unfortunately, the exact relationship between the quantities 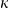 and 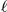 in the transmission network and the contact network has not yet been described. However, since the transmission network is a subgraph of the contact network, it is feasible to assume that contact networks that display long or short mean shortest paths also result in transmission networks with long or short mean shortest paths, respectively, and contact networks that have large or small mean excess degrees result in transmission networks with large or small mean excesss degrees, respectively.

### Sampling robustness

Up to this point we have only considered the case where the full transmission network is known and we can thus infer the average phylogenetic tree of the disease outbreak. It is clear, however, that in the real world we only have access to a limited subset of leaves from a phylogenetic tree. It is thus necessary to study the robustness of the tree shape under random sampling of leaves.

**Figure 4:**
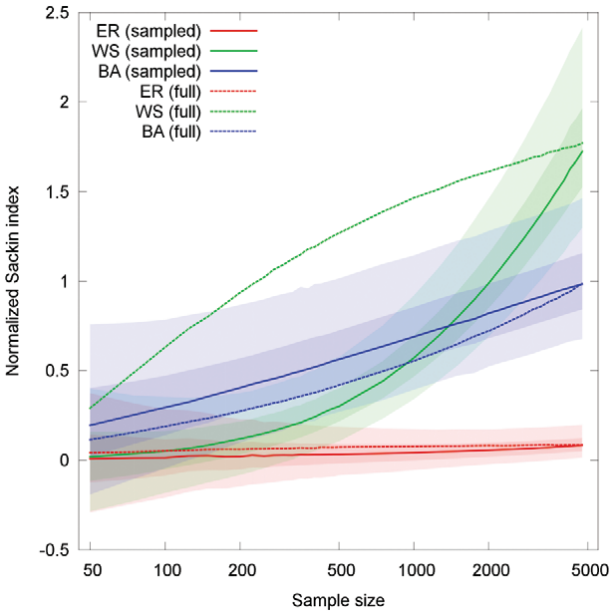
Effect of random sampling on tree balance for infection trees of an ER graph (red), WS graph (green) and BA graph (blue). The mean degree and the transmissibility are the same for all networks: 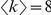 and 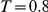. The rewiring probability for the WS is 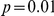. The solid lines show the median over 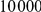 simulations and the light and dark shaded areas the 95% and 50% quantiles respectively. The dotted lines show the median normalized Sackin index for fully sampled trees of size equivalent to the sample size.

**Figure 5:**
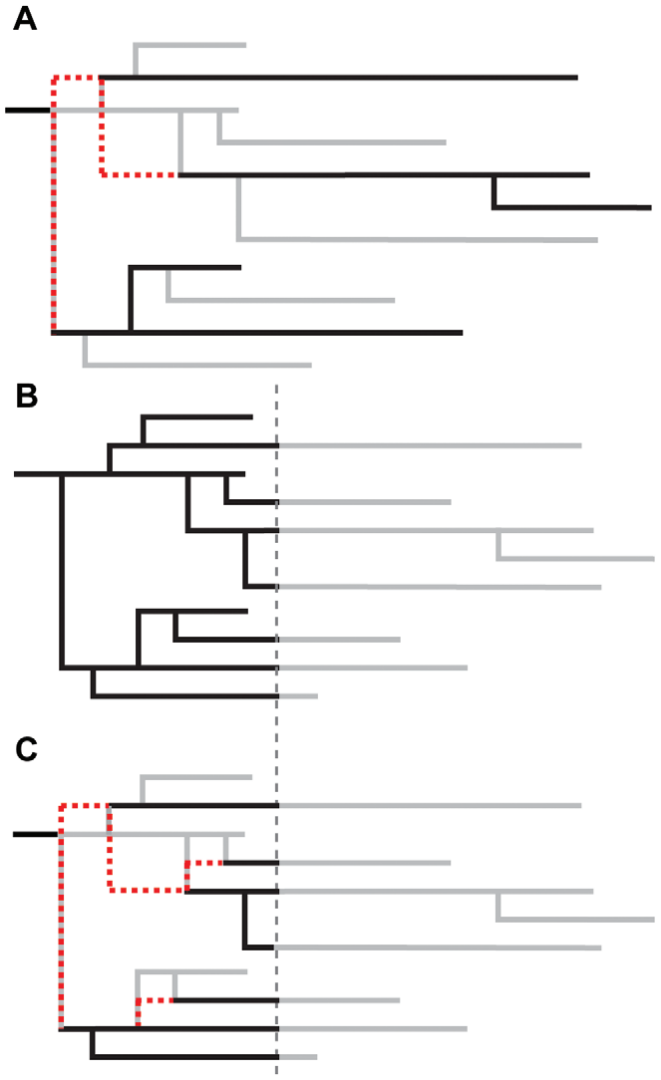
Sampling schemes of tree leaves. The grey lines represent the full transmission tree. The red dashed lines are the reconstructed coalescent events of the sampled branches. (A) Random sampling: branches are randomly selected from the complete tree. (B) Sampling up to time 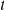: all transmission events that happened before time 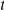 are kept. (C) Sampling at time 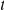: only branches alive at a given time 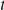 are kept and the coalescent events are reconstructed.

Figure 4 shows the imbalance of the tree as a function of the number of sampled lineages. All non sampled branches are pruned from the tree and the sampled branches are joined together at their last common ancestor to create the sample tree (see Figure 5A). For small enough sampling sizes (around 1%) the ER and WS graphs become indistinguishable, indicating that the imbalance is driven by the finer structures of the tree, rather than the backbone. The imbalance of the BA network converges much slower to that of the ER network.

Figures 5B and 5C show two schemes of time sampling for which we study the effect on tree imbalance. In the first scheme we truncate the tree at a time point 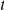 before the end of the epidemic (Figure 5B). This corresponds to the situation where samples of all individuals in an ongoing epidemic are available. In the second scheme, we use only those sequences from individuals that are infectious at a time point 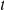 and exclude sequences from individuals who are no longer infectious or have died before 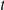 (Figure 5C). This corresponds to a snapshot of an epidemic.

**Figure 6:**
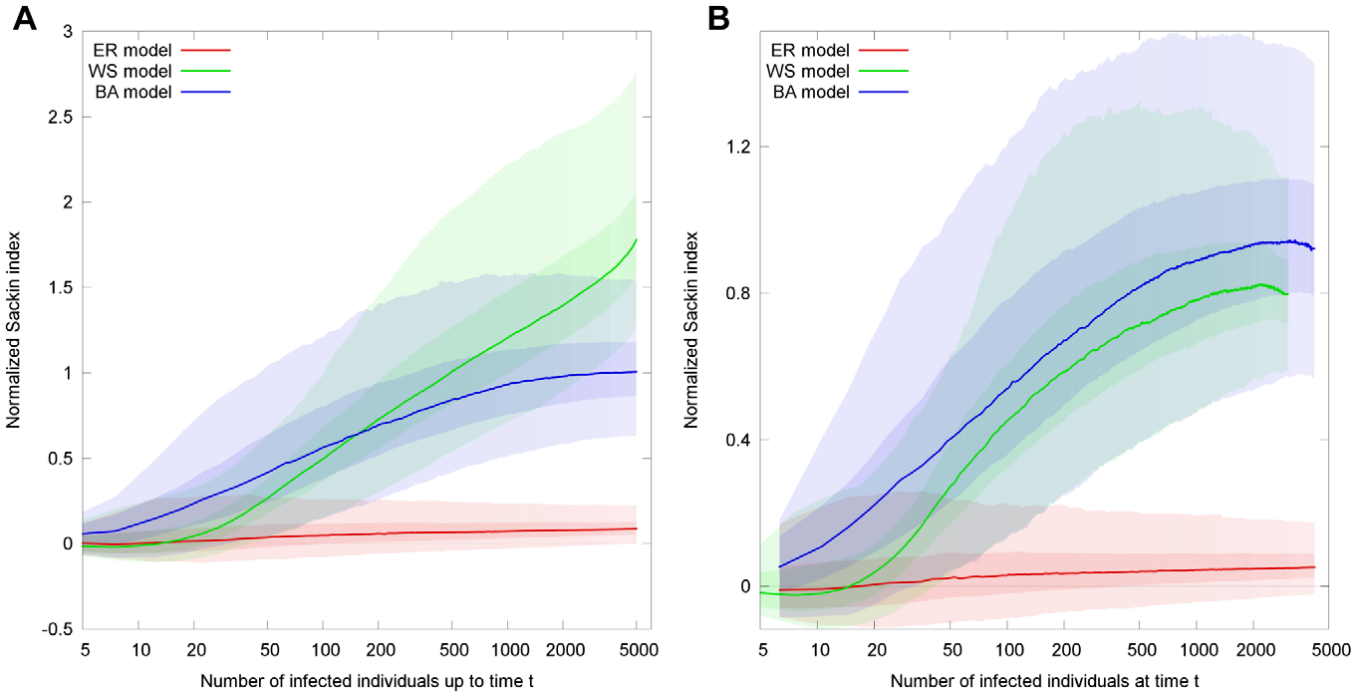
Time evolution of the normalized Sackin index, 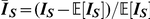 for the networks generated by the ER (red), BA (blue) and WS (green) model respectively. For all models, the mean number of neighbors 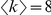 and the total population size 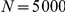. The epidemic parameters are 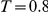. For the WS model, the rewiring probability is 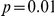. 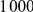 epidemics on different networks were simulated for each of the three networks models. (A) Tree imbalance when all individuals that have been infected prior to the time 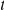 are included in the tree. While both the ER and BA models saturate at a certain value of the normalized Sackin index, the WS model continues to grow exponentially with new infected individuals. (B) Only those individuals which are infectious at time 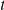 are included in the tree.

In Figure 6A, we observe that tree balancedness saturates at a certain value for ER and BA models, even before the epidemic has stopped. In the case of the WS model, tree imbalance continues to grow exponentially until the last individual has been infected. This indicates that in the ER and BA models, the early stages of the epidemic contribute more strongly to tree imbalance. In contrast, in the WS model the late stage infections contribute more strongly than the early stage infections. This is consistent with the observations made in the case of random sampling, since random sampling tends to destroy the tree structure towards the tips of the tree, while conserving the structure towards the root of the tree. This differentiation can no longer be observed when a snapshot of the epidemic is used to create the tree (Figure 6B).

The two schemes of time sampling are studied here because they are characteristic for data sampling in different biological contexts. The first scheme reflects the typical situation for real epidemics for which sequence information is sampled over a broad time window. The second scheme is more applicable to phylogenetic trees based on pathogen populations from within an individual host. While we have concentrated so far on the inference of epidemiological contact structure from phylogenetic trees, we note that our approach can also be used to study the imbalance of within-host trees, which may result from spatial structure or compartmentalization. Both these schemes are idealizations of available real data. In most situations the sample structure will in fact be a combination of one of the two time sampling schemes and random sampling as discussed in the previous section.

### HIV epidemic

**Figure 7:**
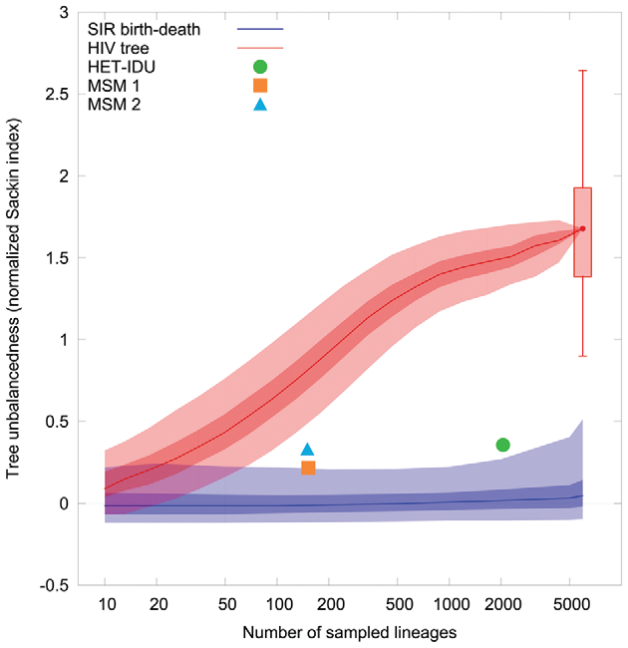
Tree imbalance of the Swiss HIV phylogenetic tree and 100 bootstrap trees (candlestick). The solid red curve shows the behavior of the normalized Sackin index for a tree with randomly sampled leaves from the complete HIV tree. The blue curve is the normalized Sackin index of an epidemic in a susceptible population displaying random mixing. The size of the susceptible population is chosen uniformly from the interval 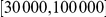. The total epidemic size is chosen uniformly from the interval 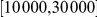. The removal rate 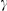 was chosen to be 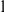 and transmission rate such that 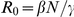. We simulated 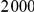 outbreaks for each of the sample sizes of 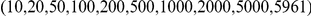. The light shaded areas show the 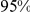 credible intervals, the dark shaded areas the 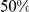 credible intervals. The individual data points are the values of the normalized Sackin index for the three largest transmission clusters: heterosexuals/intravenous drug users (HET/IDU) and two men having sex with men (MSM) clusters.

Above we demonstrated that contact structure can result in strongly unbalanced trees. Here we investigate whether real epidemics also result in unbalanced trees. To this end we examine the imbalance of a phylogenetic tree constructed from 5961 patient sequences of the Swiss HIV cohort study [33] (see Figure 7).

Since SIR dynamics with low 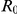 (i.e. small mean degree 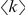 or transmissibility 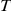 close to the critical value 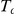) can potentially also generate strongly unbalanced trees, we compare the imbalance of the HIV tree to an SIR epidemic with random mixing and an 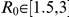, corresponding to the range of realistic 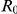 that has been estimated for the HIV epidemic in Switzerland [34]. The sampled individuals cover 30–40% of all Swiss HIV infected individuals and we therefore restrict the total epidemic size to the range 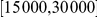. It has been argued that the HIV epidemic is still in the exponential stage in developed countries [35]. However, because saturation of an epidemic also causes increased imbalance, we make the conservative assumption that the total population is finite and can be equal to the current epidemic size. We take the range of possible population sizes to be 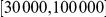. As a null model, we use a likelihood-free test of departure from random mixing based on [36]. We repeatedly sample parameters uniformly from the intervals above and simulate an epidemic outbreak using these parameters under the assumption of random mixing. We then randomly sample between 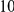 and 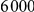 individuals from the simulated tree and calculate the normalized Sackin index of the resulting subtree (blue line and shaded areas in Figure 7). We compare this to subtrees with identical number of sampled individuals from the HIV tree from [33] (red line and shaded areas in Figure 7; see Figures S3, S4 and S6 for an analysis using an alternative imbalance measure, as well as a more detailed view of the effect of individual parameters on tree imbalance).

Comparing the HIV tree with an SIR epidemic with equal number of individuals connected by random mixing shows that the HIV tree exhibits strong imbalance. The normalized Sackin index of the HIV tree is 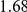 with a minimum/maximum of 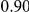/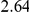 based on 100 bootstrap trees constructed from sequences with the amino acid positions resampled. The range of values of the normalized Sackin index of the HIV tree as well as the bootstrap trees is outside the 95% confidence interval for the SIR model, implying that the imbalance of the HIV tree is statistically highly significant.

One important component of contact structure in the HIV epidemic is the preferential transmission within transmission groups (such as heterosexuals, intravenous drug users, and men having sex with men) [33]. Subepidemics occurring within these transmission groups are therefore expected to show decreased levels of imbalance. Indeed, calculating the Sackin index for the three largest transmission clusters [33] reveals much more balanced trees in these subepidemics (see Figure 7). However, the observed level of imbalance is still significant, suggesting that contact structure is present even within these transmission groups. As we pointed out above, the imbalance in the SIR model increases with 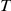 approaching 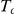. Therefore, the significance of the imbalance of the subepidemics depends on the choice of 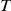 and thus 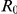.

In summary, our analysis of the HIV tree reveals substantial imbalance in the entire epidemic, possibly extending to the subepidemics, which is consistent with what would be expected from our knowledge of HIV transmission.

## Discussion

In this paper, we have studied the effect of different classes of contact networks to model SIR type epidemics. We show that simulations of epidemics on networks with non-random contact structure result in transmission trees with topologies that exhibit strong differences from tree topologies that would be obtained under the assumption of random mixing. Measures of tree imbalance such as the (normalized) Sackin index can be used to reveal such differences and to quantify the statistical significance of departure from models assuming random mixing.

Epidemiological properties, such as rate of spread or probability of outbreak, are known to depend sensitively on contact structure. If appropriate genetic data are available, then the approach presented here allows testing whether an epidemic may be appropriately modeled by standard models assuming random mixing. Conversely, if one is interested in phylogenetic tree structure of infectious pathogens, then knowledge of the contact structure in the host population will be important for the correct interpretation of the tree topology. Such contact structure may be on a coarse grained level (e.g. in between cities for human infectious diseases) or on a fine grained level (e.g. contact patterns between individuals). The level at which a phylogenetic tree is able to resolve any contact structure depends on the rate of evolution of the pathogen. In cases such as HIV, where the rates of evolution are high enough to result in substantial genetic differences between virus populations of individual hosts, a phylogenetic tree may reveal contact structure down to the individual level. Indeed our analysis of the phylogenetic tree underlying the Swiss HIV epidemic provides evidence for non-random contact structure on the population level as well as the individual level. A considerable part of the imbalance of the HIV tree is likely attributable to a high-level contact structure that arises from preferential transmission within transmission groups (such as heterosexuals, intravenous drug users, and men having sex with men). An analysis of the imbalance of subtrees corresponding to individual transmission groups, however, also reveals a signal indicative of non-random contact structure within these transmission groups.

The importance of contact structure for epidemiological processes has been clearly demonstrated by a large number of theoretical studies [2], [4], [5], [8], [10], [11], [37]. This has sparked considerable interests in determining the contact structure that underlies the transmission of different pathogens [12], [13], [16]. The determination of such contact networks is fraught with difficulties. Contact networks based on patient interviews may suffer from incompleteness, inaccuracy and in some cases also lack of reliability of patient information. Contact networks derived from devices that measure physical proximity often do not have sufficient spatial resolution or may neglect to account for important pathogen specific factors.

Our method takes a first step to infer contact structure from genetic data. In comparison to the other methods mentioned above it has the advantage of being based on data that are readily available for many important pathogens. Moreover, our approach focuses only on those contacts that have led to transmission rather than other contacts between individuals that may be irrelevant for the spread of the epidemic. The method presented here allows testing for deviations from the assumption of random mixing. It is possible to extend the approach to test departure from BA or WS networks or other network models (e.g. configuration model networks with specific degree distributions), equivalent to the Swiss HIV epidemic (see Results). Thus our approach is able to distinguish between different types of contact networks in a statistical sense, but it does not yield the actual contact network that underlies the epidemic. The choice of null model will strongly depend on the epidemic studied. Different diseases have different transmission routes and thus a contact that is relevant for transmission for one disease may not be relevant for transmission of another disease.

Our method is based on imbalance, which is only a crude measure of tree topology. We have shown that imbalance cannot distinguish between BA and WS networks in our case. We expect that using other independent measurements of tree topology can reveal further information on the structure of the underlying contact network. Branch lengths are one such measure [22], [27]. In simulated epidemic outbreaks, where the exact waiting times between infection and recovery events are known, branch lengths can be used together with tree imbalance to distinguish between ER, BA and WS models (Figure S7). It is important to note that maximum likelihood analyses typically provide trees where branch lengths represent evolutionary time. The branch length statistic used in Figure S7 requires edge lengths in calender time. In order to obtain accurate branch length estimates in calender time rather than evolutionary time, we need to allow for the observed variation in evolutionary rates across branches, such as relaxed clock models [38]. Due to the model complexity, these analyses are typically done in a Bayesian MCMC framework which does not converge for datasets of our size using the current implementation [39]. Thus the reliability of branch length estimates in our reconstructed phylogenies is questionable and this measure should only be used when confidence in branch lengths of the reconstructed tree is very high.

The imbalance of the reconstructed phylogenies depends on the the genetic data used. Sampling biases at the genetic level can result in a strongly unbalanced tree [27], even if the underlying population is randomly mixed. This sampling bias will be reflected in the inferred contact structure. In this sense, if connected subsets of the population are more densely sampled than others, the resulting contact structure will show that these individuals are much more highly interconnected than the other individuals that belong to those sub-populations that are only sparsely sampled. This can be both advantageous as well as disadvantageous, depending on what the contact structure should reflect. If the sampling of genetic data is high in those sub-populations where we require high resolution, then the inferred contact structure will be representative of this sub-population, but not of those that are poorly sampled. Our method would therefore reject an epidemic model of contact structure where all individuals are equally likely to be interconnected for one where some individuals are highly connected (i.e. those from the densely sampled sub-population) and others are weakly connected (i.e. those from the sparsely sampled sub-population).

We also note that the approach presented here can be applied to the analysis of phylogenetic trees based on pathogen populations within an infected individual. The models for the dynamics of pathogen populations within an infected individual typically are also based on the assumption of random mixing and our approach would allow to test whether this assumption is fulfilled. For phylogenetic trees based on within-host data imbalance would likely reflect a compartmentalization of pathogen replication and could thus provide important insight into mechanisms of pathogenesis.

## Materials and Methods

### Ethics statement

The Swiss HIV cohort study was approved by individual local institutional review boards of all participating centers (www.shcs.ch). Written informed consent was obtained for each SHCS study participant.

### Deterministic SIR/SI model

We consider a disease spreading amongst a susceptible population that displays susceptible-infected-removed (SIR) type dynamics [1], [40]. In the limit of large population size and random mixing the model can be described by the simple system of differential equations
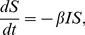
(2)

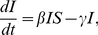
(3)

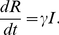
(4)

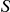
, 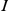 and 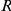 are the number of susceptible, infected, and removed individuals in each compartment at time 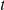. Here, 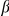 is the rate of transmission per contact between a susceptible and infected individual and 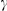 is the removal rate of infected individuals. In the context of a network the transmissibility 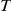 is the probability that an individual will transmit the disease across a single contact over the whole duration of the epidemic. This can be calculated from 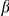 and 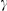 by averaging over the distribution of waiting times for transmission and recovery. For a given recovery time 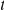, the probability that transmission occurs before the individuals recovers is given by 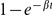. Thus, if the recovery times are exponentially distributed [5],
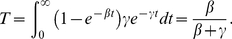
(5)The basic reproductive ratio 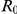 is the number of secondary infections caused by an infected individual placed into a wholly susceptible population (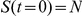) [1]. In fully mixed populations, an epidemic can occur when 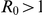. Here, 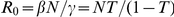. In non-homogeneous populations this threshold also depends on the contact structure. For networks generated by the configuration model [41], [42], i.e. random contact networks with a given degree distribution, the expected total number of second neighbors (neighbors of my neighbors) is given by 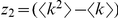, where 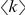 and 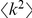 are the first and second moments of the degree distribution [43]. Then 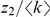 is the average number of nodes two steps away per neighbor. Thus the expected number of secondary infections per infected individual is 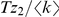. For an epidemic to occur 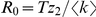 must be greater than 1 or 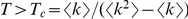 [2], [5]. When the population additionally displays community structure (such as clustering and modularity) this threshold changes again. For example, the Watts-Strogatz model incorporates local connectedness by starting with a regular network where every node is connected to a fixed number of close neighbors. Then, each connection is rewired to a randomly chosen node with a certain probability, thus creating shortcuts in the contact network [31]. In this case the threshold for an epidemic outbreak also depends on this rewiring probability [37].

Since we are not interested in the exact values of the parameters, we can choose 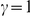 by rescaling 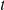 without loss of generality. Furthermore, it should be noted that as 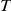 approaches 1, 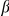 gets much larger than 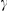. Hence, the SIR model with large 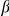 effectively reduces to an SI model.

### Simulation of transmission trees

In order to simulate transmission trees of epidemics occurring in heterogeneously connected populations, a C_++_ implementation of Gillespie's Next-Reaction Method was used [44]. At the beginning of the simulation a single node is infected and a recovery time 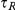 is sampled from the distribution of recovery times, 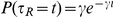. Each of the node's susceptible neighbors is then infected after a time 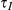 chosen from the distribution of infections times, 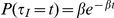. If the infection time is shorter than the recovery time, the link is activated and the node is infected at time 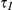. The procedure is then repeated for each newly infected node. In case a node is scheduled to be infected by multiple neighbors, the earliest infection takes priority. By keeping track of who-infects-whom, each epidemic outbreak yields an infection tree.

### Network generation

We study three different network models: (a) the Erdös-Rényi (ER) random graph [29], (b) the Barabási-Albert (BA) graph [30] and (c) the Watts-Strogatz (WS) graph [31]. In the ER random graph every individual is connected to every other individual with a certain probability 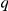. This results in a graph with a Poissonian degree distribution with mean number of neighbors 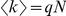. The BA graph is constructed by preferential attachment. Each node is sequentially added to the graph and attached to 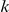 neighbors, where nodes that already have many neighbors have a higher probability of being connected to the new node. This results in a degree distribution with a power-law tail. Such graphs are often referred to as scale-free [30]. Finally, WS graphs start out with a ring lattice, in which every node is connected to its 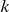 nearest neighbors. Each link is then updated with probability 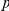 in such a way that one end of the link is rewired to a randomly chosen node. Thus the node that loses the link decreases its degree by one and the node that the link is rewired to increases its degree by one. This process introduces shortcuts in the graph (i.e. decreases the mean shortest path) [31]. For 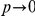 the graph has strongly connected communities. For 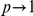 all links are randomly assigned and the graph is similar to the ER graph with the same mean number of neighbors (equal number of edges) [42]. For intermediate values of 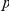, the graphs often display both strong community structure and short path lengths, which are characteristics of small-world graphs [31].

### Phylogenetic tree shape

The shape of a phylogenetic tree is described in part by its imbalance. Here, we use the Sackin index as a measure of imbalance [45], because of its analogy to path lengths in graph theory. The Sackin index is defined as follows: Let the distance 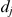 of a leaf 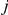 be the number of internal nodes that need to be traversed when following the path from the root of the tree to a leaf 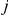. Then the Sackin index is the sum of all such paths,
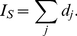
(6)


When considering transmission trees, it is important to differentiate between two cases: The first case considers the complete transmission trees of an epidemic outbreak. This is essentially equivalent to a birth/death process. From the perspective of an individual, death corresponds to removal from the infectious class or the depletion of all its susceptible neighbors. In either case that individual can no longer infect anyone else. Thus the transmission trees have branches that do not all survive until the end of the epidemic.

In the second type of tree, all lineages are extant at the end of the epidemic. Such a transmission tree could be generated by an SI-type epidemic in an infinite size population where each individual can infect every other individual. These trees are generated by the Yule model.

The expected value of the Sackin index for a given number of leaves 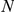 under the Yule model is given by [26],
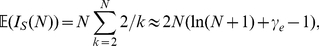
(7)with Euler's constant 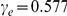.

An exact expression for the expected value of the Sackin index is not known in the case where some lineages die before the end of the epidemic. However, it can be assumed that this will in general result in slightly more unbalanced trees.

Since the expected value of the Sackin index increases with tree size, we introduce a normalized Sackin index defined by
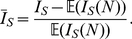
(8)

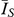
 measures the relative deviation of the tree imbalance from what would be expected for an SI epidemic (or SIR with 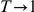).

## Supporting Information

Figure S1 An example illustrating the difference between (A) a contact network, (B) a transmission network where one initially infected individual (red node) causes a disease to spread along the red edges and (C) the resulting phylogenetic tree if the order in which the infections took place is known. This distinction has recently also been illustrated by Welch et al., Viruses 2011.
(TIF)

Figure S2 Cartoon of an epidemic outbreak on two idealized contact networks: (A) A chain representing an extreme case of a WS network without long-range connections. (B) An extreme case of preferential attachment, where one single center node is connected to all other nodes. The branching points in the tree represent infection events and the colors indicate the lifespan of the corresponding node in the network. Branch lengths have no significance in these cartoons.
(TIF)

Figure S3 Density plot of two imbalance measures (x-axis: normalized Sackin index; y-axis: s-index) for 10 000 trees generated under the SIR model with random mixing. The parameters are chosen the same as for figure 7 in the main text: *R_0_* = 2.14; total susceptible population: *N* = 40 000; maximum epidemic size: *M* = 20 000; number of sampled individuals: *n* = 5 961. The colors in the scatter plot indicate the frequency at which the SIR model resulted in a tree with imbalance measures in the given range. The large red dot indicates the maximum likelihood HIV tree and the small red dots are 100 bootstrap trees. For the normalized Sackin index all HIV trees lie outside of the distribution of SIR trees (to the right in the *x*-direction). The s-index, however, is unable to reject the SIR model for the HIV data. Only a small number of trees (437/10 000) generated by this process are rejected by the Sackin index test while not being rejected by the s-Index test (data lies outside the 95% interval).
(TIF)

Figure S4 s-Index test for sub-trees of the HIV tree. *y* = 0 indicates the 97.5-th quantile of the s-Index for trees generated by the SIR model. Positive *y*-values indicate a rejection of the SIR model. MSM1 and MSM2 are the two largest MSM (men-having-sex-with-men) transmission groups in the HIV tree. HET-IDU is the largest heterosexual/intravenous-drug-user transmission group. Thus, despite the s-index not being able to reject the SIR model for the full HIV tree, sub-samples of the HIV tree can reject the SIR model using the s-index.
(TIF)

Figure S5 Blue: Normalized Sackin index for networks generated by the WS model for varying rewiring probability, transmissibility *T* = 0.6, *K* = 8 and *N* = 5000. Green: Normalized Sackin index for a graph generated by the configuration model with degree sequence equal to the WS model. The light and dark shaded areas represent the 95 and 50 percent confidence intervals.
(TIF)

Figure S6 Distribution of tree imbalance statistics for trees generated under the SIR model with random mixing for different parameter combinations and *R*_0_. The colors in the scatter plot indicate the frequency at which the SIR model resulted in a tree with imbalance measures in the given range. The red dot indicates the real HIV tree. The normalized Sackin index rejects the SIR model for all choices of *R*_0_, total susceptible population size and maximum epidemic size. (A) Total susceptible population: *N* = 40 000; maximum epidemic size: *M* = 20 000; number of sampled individuals: *n* = 5 961. (B) Total susceptible population: *N* = 30 000; maximum epidemic size: M = 20 000; number of sampled individuals: *n* = 5 961. (C) Total susceptible population: *N* = 40 000; maximum epidemic size: *M* = 10 000; number of sampled individuals: n = 5 961.
(TIF)

Figure S7 Tree imbalance (normalized Sackin index) plotted against the mean external branch length divided by the mean internal branch length at different values of transmissibility. This figure complements figure in the main text. The different colors and point shapes are networks generated by the three network models: ER (green circles), BA (red triangles), WS (blue diamonds). In all panels, *N* = 5000 and γ = 1. The connectivity for the WS model is *p* = 0:01. For each value of *T*, 2 000 simulations were performed per network model. Only those simulations where an outbreak occurred (epidemic size >.05*N*) are plotted. For those values of *T* for which tree imbalance cannot distinguish between network models (e.g. at *T* = 0.2 the ER and BA model overlap in tree imbalance), information about branch lengths can potential give an additional resolution.
(TIF)

Text S1 Detailed description of the methods. (A) Derivation of the Sackin index for a random network generated by the configuration model. (B) Factors leading to imbalanced trees. (C) Comparison of different tree balance statistics. (D) Testing the HIV tree using different tree imbalance statistics. (E) Effect of path length on tree imbalance for WS networks.
(PDF)

Text S2 Detailed analysis of population size N and mean number of neighbors *k* on tree imbalance.
(PDF)
