# Supplementary figures and images for: Inferring Epidemic Contact Structure from Phylogenetic Trees
Source: PLoS Comput Biol. 2012 Mar 8;8(3):e1002413. doi: 10.1371/journal.pcbi.1002413 (PMC3297558; doi:10.1371/journal.pcbi.1002413)

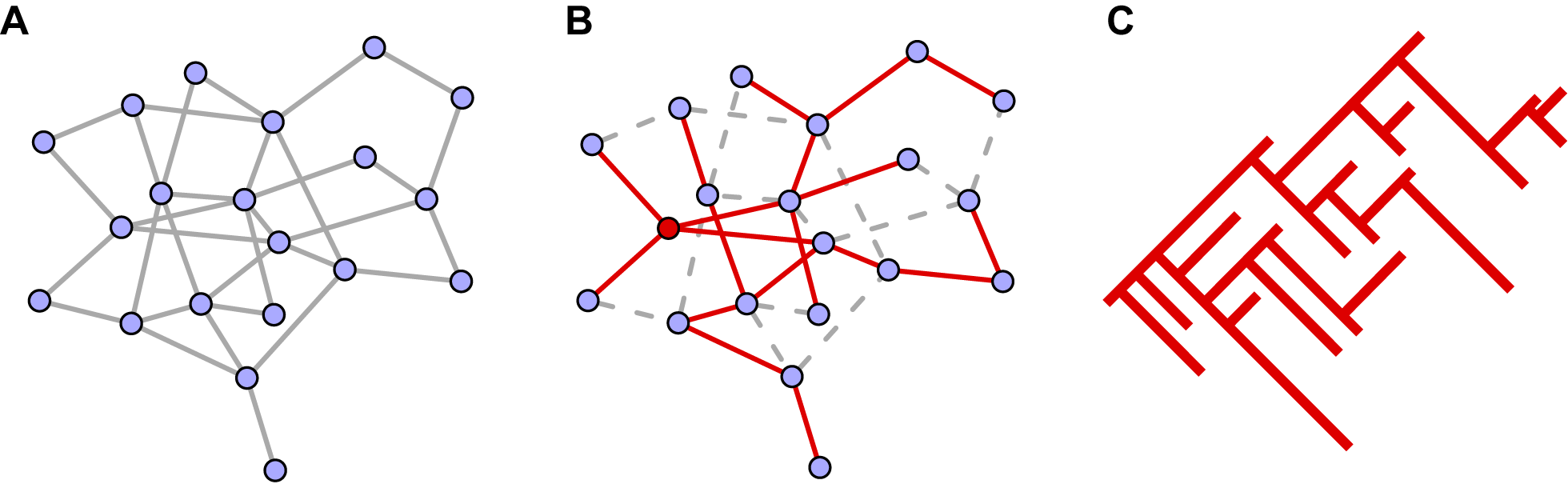

Supplement: Figure S1 — An example illustrating the difference between (A) a contact network, (B) a transmission network where one initially infected individual (red node) causes a disease to spread along the red edges and (C) the resulting phylogenetic tree if the order in which the infections took place is known. This distinction has recently also been illustrated by Welch et al., Viruses 2011. (TIF) [file pcbi.1002413.s001.tif]

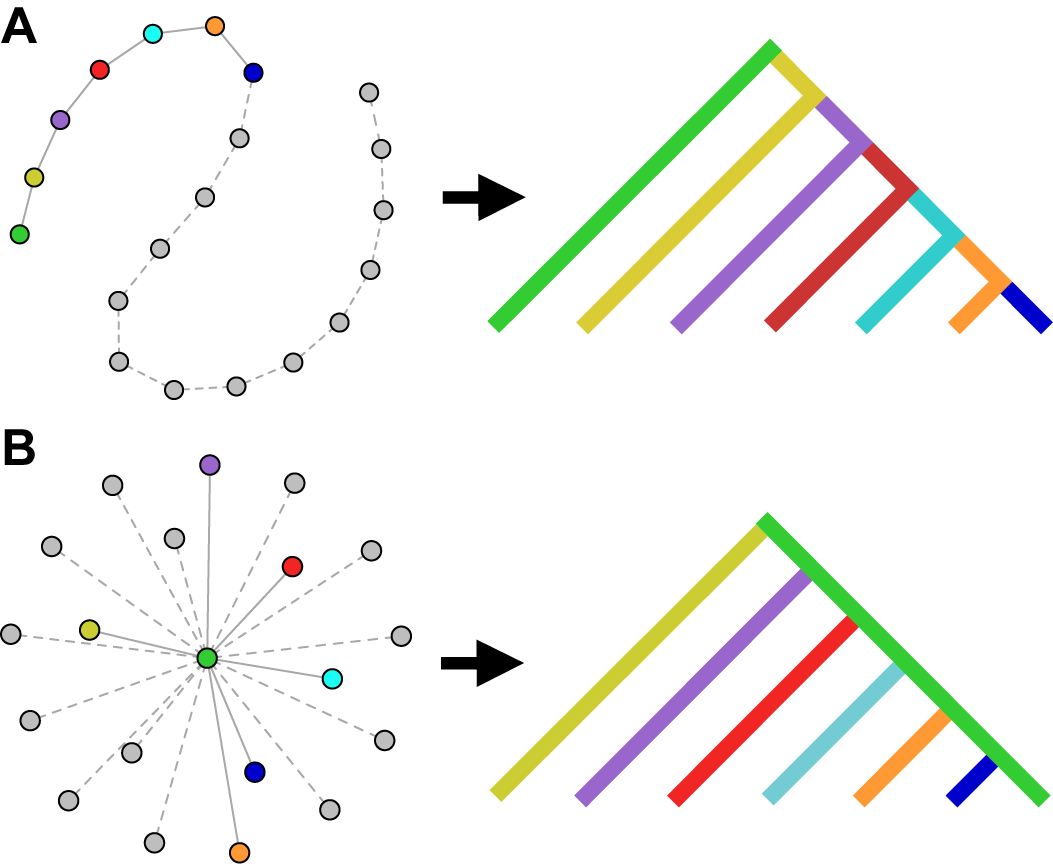

Supplement: Figure S2 — Cartoon of an epidemic outbreak on two idealized contact networks: (A) A chain representing an extreme case of a WS network without long-range connections. (B) An extreme case of preferential attachment, where one single center node is connected to all other nodes. The branching points in the tree represent infection events and the colors indicate the lifespan of the corresponding node in the network. Branch lengths have no significance in these cartoons. (TIF) [file pcbi.1002413.s002.tif]

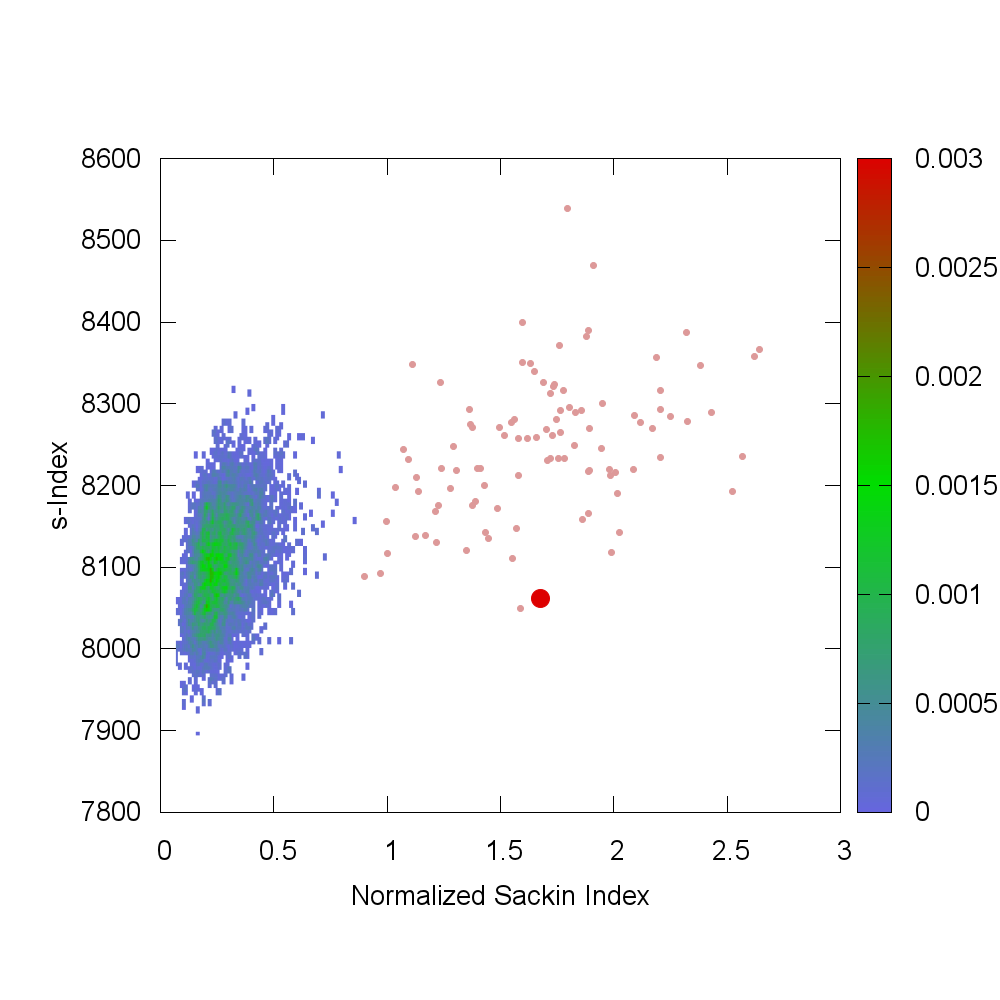

Supplement: Figure S3 — Density plot of two imbalance measures (x-axis: normalized Sackin index; y-axis: s-index) for 10 000 trees generated under the SIR model with random mixing. The parameters are chosen the same as for figure 7 in the main text: R0 = 2.14; total susceptible population: N = 40 000; maximum epidemic size: M = 20 000; number of sampled individuals: n = 5 961. The colors in the scatter plot indicate the frequency at which the SIR model resulted in a tree with imbalance measures in the given range. The large red dot indicates the maximum likelihood HIV tree and the small red dots are 100 bootstrap trees. For the normalized Sackin index all HIV trees lie outside of the distribution of SIR trees (to the right in the x-direction). The s-index, however, is unable to reject the SIR model for the HIV data. Only a small number of trees (437/10 000) generated by this process are rejected by the Sackin index test while not being rejected by the s-Index test (data lies outside the 95% interval). (TIF) [file pcbi.1002413.s003.tif]

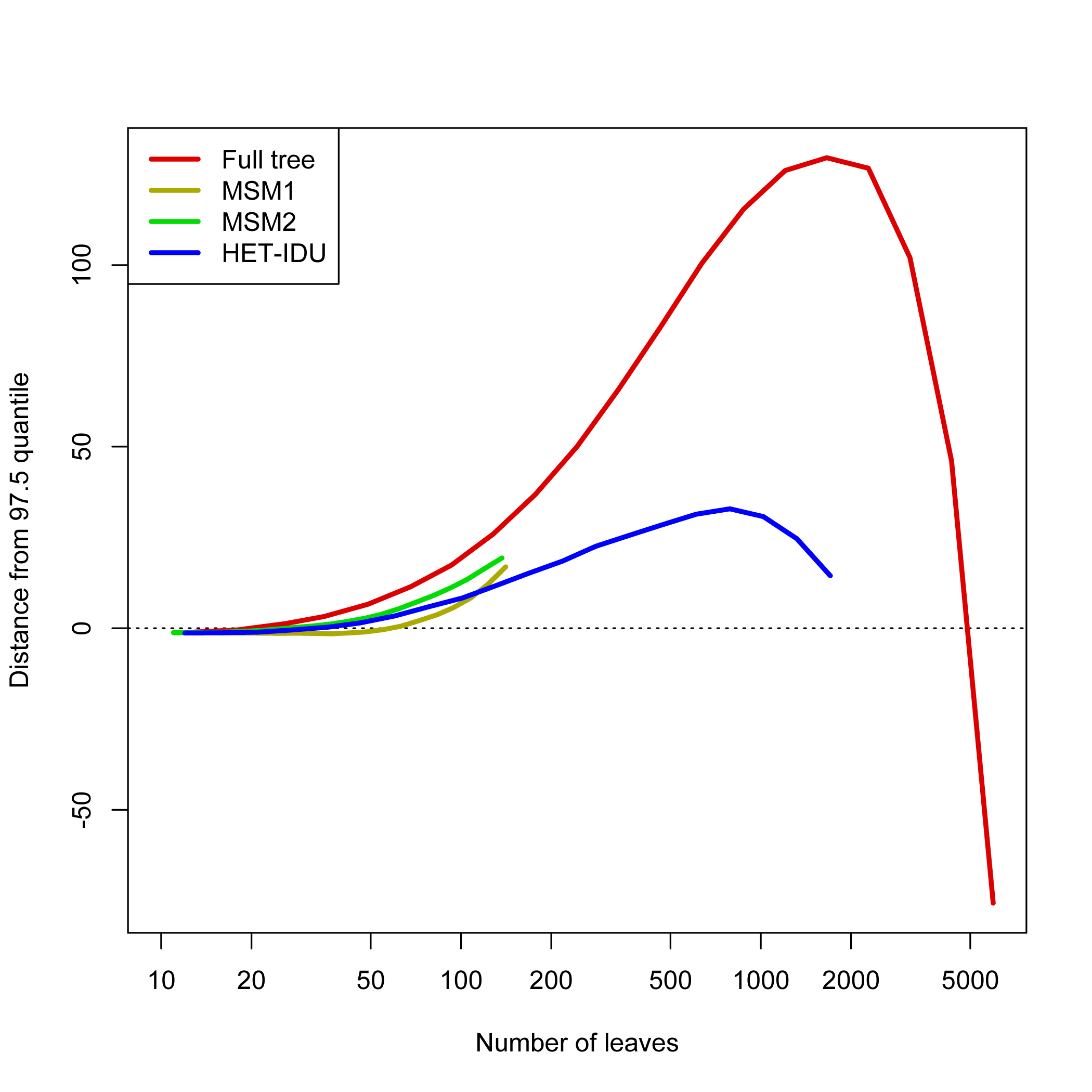

Supplement: Figure S4 — s-Index test for sub-trees of the HIV tree. y = 0 indicates the 97.5-th quantile of the s-Index for trees generated by the SIR model. Positive y-values indicate a rejection of the SIR model. MSM1 and MSM2 are the two largest MSM (men-having-sex-with-men) transmission groups in the HIV tree. HET-IDU is the largest heterosexual/intravenous-drug-user transmission group. Thus, despite the s-index not being able to reject the SIR model for the full HIV tree, sub-samples of the HIV tree can reject the SIR model using the s-index. (TIF) [file pcbi.1002413.s004.tif]

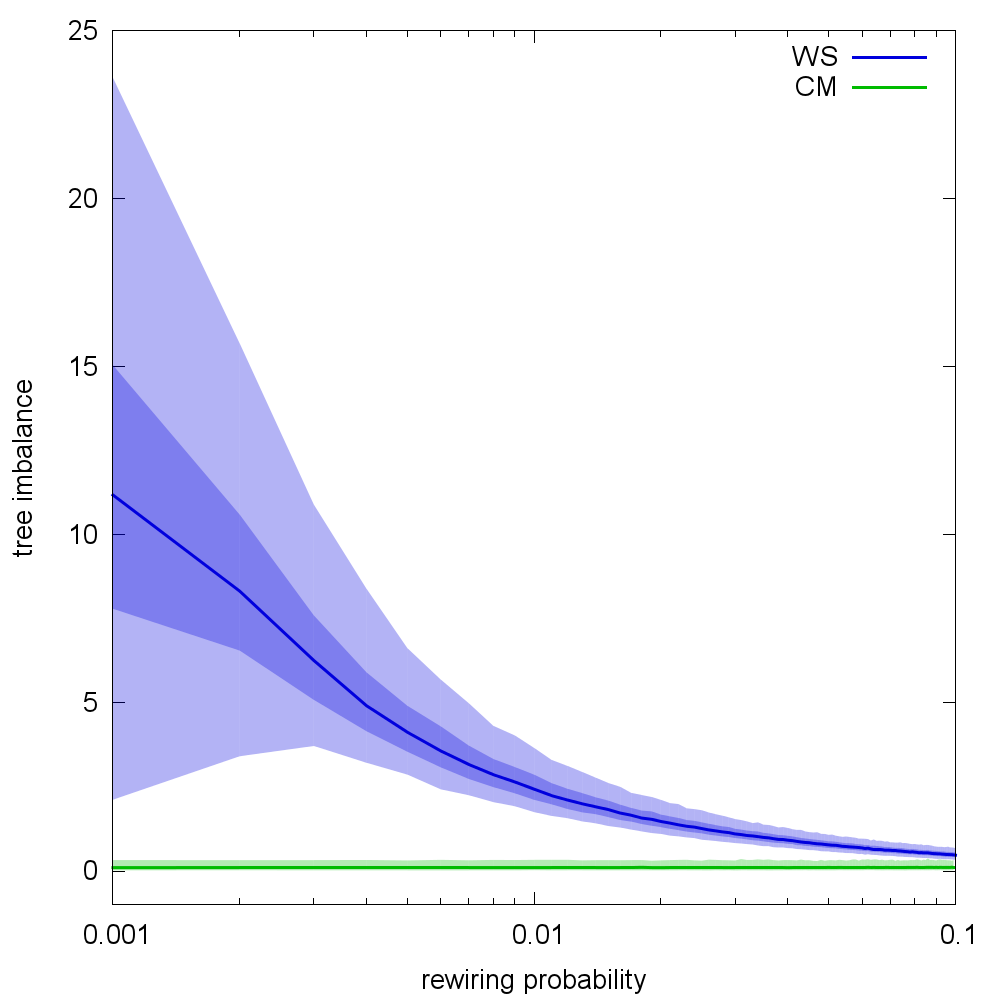

Supplement: Figure S5 — Blue: Normalized Sackin index for networks generated by the WS model for varying rewiring probability, transmissibility T = 0.6, K = 8 and N = 5000. Green: Normalized Sackin index for a graph generated by the configuration model with degree sequence equal to the WS model. The light and dark shaded areas represent the 95 and 50 percent confidence intervals. (TIF) [file pcbi.1002413.s005.tif]

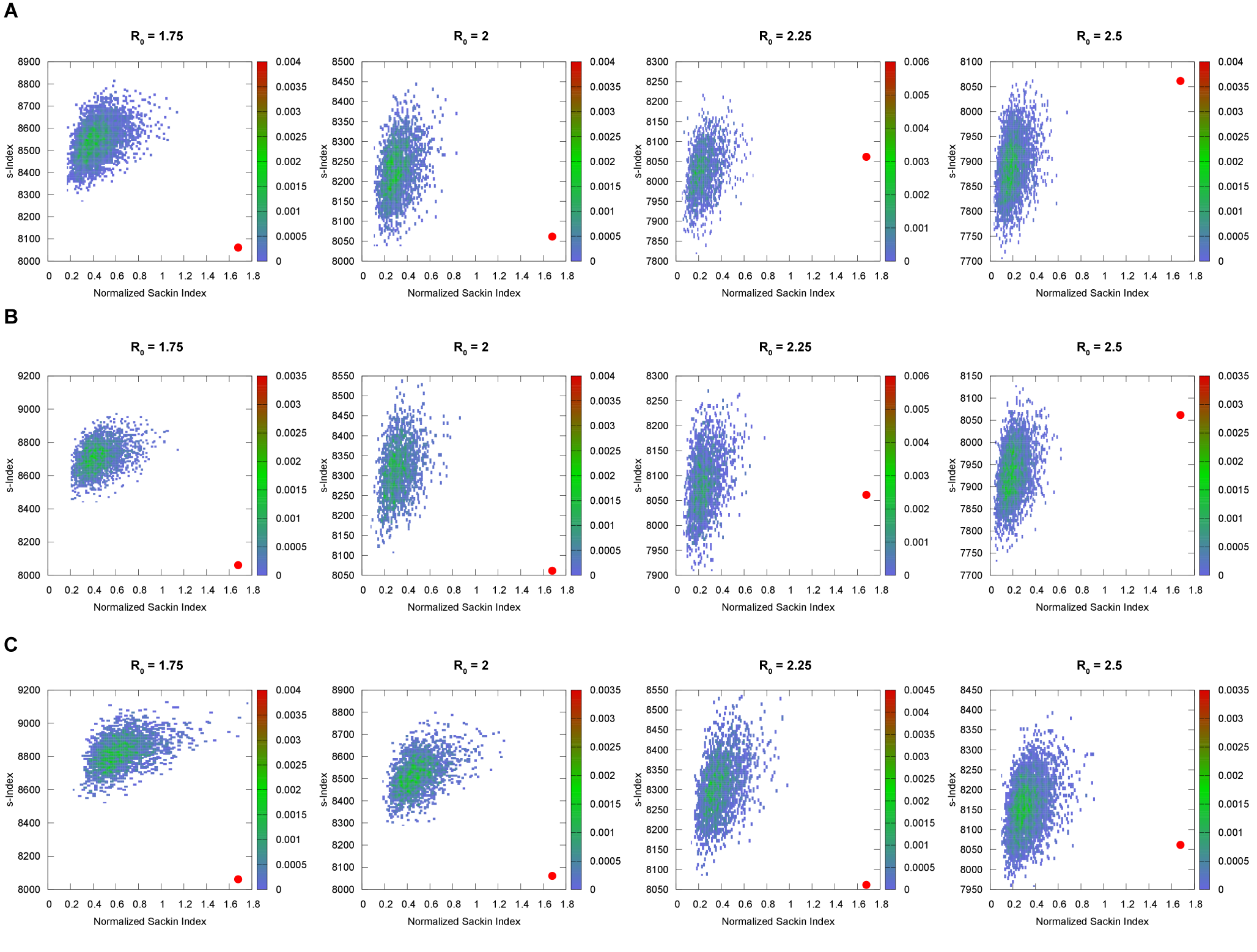

Supplement: Figure S6 — Distribution of tree imbalance statistics for trees generated under the SIR model with random mixing for different parameter combinations and R 0. The colors in the scatter plot indicate the frequency at which the SIR model resulted in a tree with imbalance measures in the given range. The red dot indicates the real HIV tree. The normalized Sackin index rejects the SIR model for all choices of R 0, total susceptible population size and maximum epidemic size. (A) Total susceptible population: N = 40 000; maximum epidemic size: M = 20 000; number of sampled individuals: n = 5 961. (B) Total susceptible population: N = 30 000; maximum epidemic size: M = 20 000; number of sampled individuals: n = 5 961. (C) Total susceptible population: N = 40 000; maximum epidemic size: M = 10 000; number of sampled individuals: n = 5 961. (TIF) [file pcbi.1002413.s006.tif]

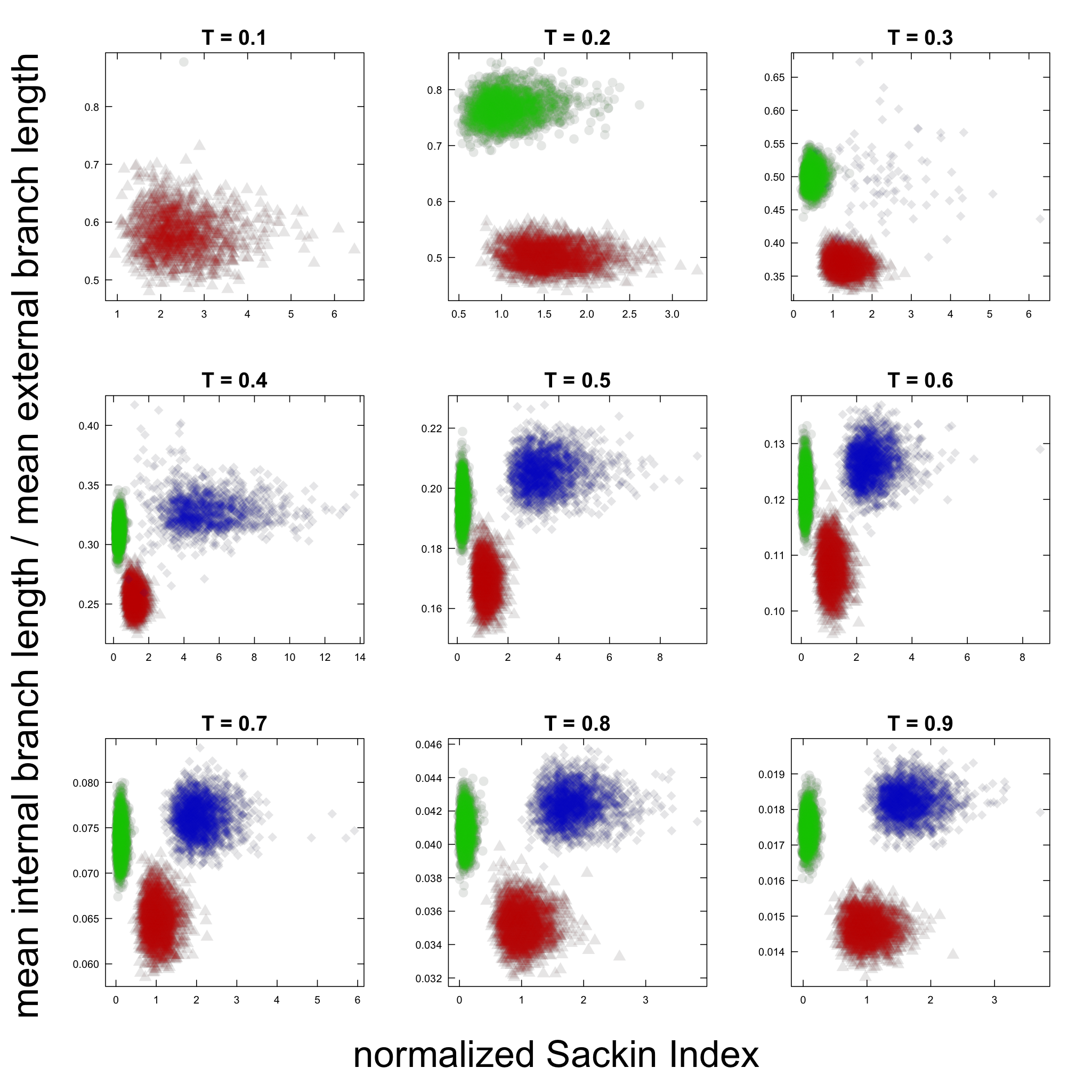

Supplement: Figure S7 — Tree imbalance (normalized Sackin index) plotted against the mean external branch length divided by the mean internal branch length at different values of transmissibility. This figure complements figure in the main text. The different colors and point shapes are networks generated by the three network models: ER (green circles), BA (red triangles), WS (blue diamonds). In all panels, N = 5000 and γ = 1. The connectivity for the WS model is p = 0:01. For each value of T, 2 000 simulations were performed per network model. Only those simulations where an outbreak occurred (epidemic size >.05N) are plotted. For those values of T for which tree imbalance cannot distinguish between network models (e.g. at T = 0.2 the ER and BA model overlap in tree imbalance), information about branch lengths can potential give an additional resolution. (TIF) [file pcbi.1002413.s007.tif]
