# Supplementary material for: Inferring Epidemic Contact Structure from Phylogenetic Trees
Source: PLoS Comput Biol. 2012 Mar 8;8(3):e1002413. doi: 10.1371/journal.pcbi.1002413 (PMC3297558; doi:10.1371/journal.pcbi.1002413)
Supplement: Text S1 — Detailed description of the methods. (A) Derivation of the Sackin index for a random network generated by the configuration model. (B) Factors leading to imbalanced trees. (C) Comparison of different tree balance statistics. (D) Testing the HIV tree using different tree imbalance statistics. (E) Effect of path length on tree imbalance for WS networks. (PDF) [file pcbi.1002413.s008.pdf]

Inferring Epidemic Contact Structure from Phylogenetic Trees: Supporting Text S1

Gabriel E Leventhal[1], Roger Kouyos[1,2], Tanja Stadler[1], Viktor von Wyl[3], Sabine Yerly[4], Jürg Böni[5], Cristina Cellerai[6], Thomas Klimkait[7], Huldrych F. Günthard[3], and Sebastian Bonhoeffer[1]

[1]Institute of Integrative Biology, ETH Zurich, Switzerland

[2]Department of Ecology and Evolutionary Biology, Princeton University, Princeton, New Jersey, United States of America

[3]Division of Infectious Diseases and Hospital Epidemiology, University Hospital Zurich, Switzerland

[4]Laboratory of Virology and AIDS Center, Geneva University Hospital, Switzerland

[5]Swiss National Center for Retroviruses, Institute of Medical Virology, University of Zurich, Switzerland

[6]Service of Immunology and Allergy, Lausanne University Hospital, Switzerland

[7]Institute of Medical Microbiology, Department Biomedicine, University of Basel, Switzerland

October 31, 2011

Contents

A Sackin index for a random network generated by the configuration model 3

B Strong local clustering as well as super-spreading leads to unbalanced trees 5

C Comparison of different tree balance statistics 6

D Testing the HIV tree using different tree imbalance statistics 7

E Path length 8

A Sackin index for a random network generated by the configuration model

The Sackin index is a measure of tree balance [1]. It is defined as

$$I_S = \sum_{i=1}^{N} d_i, \tag{1}$$

where d_i is the number of internal nodes you need to traverse when connecting leaf i to the root of the tree and N is the number of leaves. For our purposes it will be useful to rewrite this as a sum over distances in the transmission network,

$$I_S = \sum_{m=1} \sum_{j \in V(m)} D_j. \tag{2}$$

The transmission network is the subgraph of the contact network where only the edges along which an infection event occured are preserved and nodes that were not infected are removed (figure S1 and [2]). We're only interested in the expected value of I_S. If the network is of infinite size, i.e. N much larger than the epidemic size, then we can take the D_j to be independent of each other. Thus,

$$\begin{aligned} \mathbb{E}\left(I_S\right) &= \mathbb{E}\left(\sum_{m=1} \sum_{j \in V(m)} D_j\right) &(3)\\ &= \sum_{m=1} \sum_{j \in V(m)} \mathbb{E}\left(D_j\right) &(4)\\ &= \sum_{m=1} \hat{N}(m)\hat{D}(m), &(5) \end{aligned}$$

with N and D random variables that take on the number of leaves $N(m)$ that are at a distance $D(m)$ from the root and m is the distance of the corresponding node in the

infection network from the initial seed of the epidemic. $V(m)$ is the subset of node indices that are at this distance m.

If we assume that no super-infections occured then the transmission networks has a tree-like strucutre. In that case the distance $D(m)$ in the phylogenetic tree translates from the distance m in the network as,

$$\hat{D}(m) = \hat{K}(m) + \sum_{j=1}^{m} \hat{F}(j). \tag{6}$$

Here, $\hat{K}(m)$ is the average number of infections caused by a node at distance m and $\hat{F}(j)$ is the average infection order of of a node at a distance j. We can assume that there is no preferential order in which the neighbors of a given node are infected, so

$$\hat{F}(j) = \frac{\hat{K}(j) + 1}{2} \tag{7}$$

Inserting (6) and (7) in (5),

$$\mathbb{E}\left(I_S\right) = \sum_{m=1} \hat{N}(m) \left(\hat{K}(m) + \sum_{j=1}^{m} \frac{\hat{K}(j) + 1}{2} \right) \tag{8}$$

$$= \sum_{m=1} \hat{N}(m)\hat{K}(m) + \sum_{m=1} \hat{N}(m)\frac{m}{2} + \sum_{m=1} \hat{N}(m) \sum_{j=1}^{m} \frac{\hat{K}(j)}{2}. \tag{9}$$

If the contact network is sufficiently sparse, then it too will have a tree-like strucutre and short-range loops with be rare. This means that a node fails to infect one of its neighbors, that neighbor will stay uninfected for the rest of the epidemic. In this case, the number of infections caused by a node, $K(m)$, should be independent of m. Writing $\hat{K}(m) = \kappa$

and $\hat{N}(m) = z_m$,

$$\mathbb{E}(I_S) = \kappa \sum_{m=1} z_m + \frac{1}{2} \sum_{m=1} m z_m + \frac{\kappa}{2} \sum_{m=1} z_m m \tag{10}$$

$$= \kappa \sum_{m=1} z_m + \frac{\kappa + 1}{2} \sum_{m=1} m z_m. \tag{11}$$

The first term is just total number of infected nodes N. The second sum, $\sum_m z_m m$ is the sum of all path lengths from the initially infected node to all other nodes. If the initially infected node is chosen at random, then this is equal to the N times the mean path length ℓ in the infection network. Finally,

$$\mathbb{E}(I_S) = N \left(\kappa + \frac{\kappa + 1}{2} \ell \right). \tag{12}$$

B Strong local clustering as well as super-spreading leads to unbalanced trees

Figure 1A in the main text shows that both the Barabási-Albert (BA) model [3] as well as the Watts-Strogatz (WS) [4] model can result in large tree imbalance compared to the Erdős-Rényi (ER) model [5]. Here we illustrate this effect using two idealized cases of the two models.

As an extreme case of the WS model one can imagine a 1D lattice (without cyclic boundary conditions) where all nodes are only just connected to their immediate neighbors (figure S1A). If an individual at the beginning of the chain is initially infected, then this individual will just infect its immediate neighbor who will infect the next immediate neighbor until the epidemic either dies out or all individuals have been infected. This results in a maximally unbalanced tree, as branching only happens and the right-most tree branch. Conversely, a star graph can be seen as an extreme case of a preferential

attachment model, where all individuals are connected to a single center node (figure S1B). If the center node is initially infected it will continue to infect its neighbors until it either recovers or dies. This also results in a maximally unbalanced tree. As the tree shape alone cannot distinguish between the two processes, they both result in phylogenetic trees with the same Sackin index, despite having very different contact structures.

This illustration also reflects the dependence on path length and degree variance. Both networks in figure S1 have the same mean degree since the number of nodes and edges is the same in both networks. However, the star graph has a very large degree variance (one node with degree $n-1$ and $n-1$ nodes with degree 1) and a short mean path length of approximately 2. In the chain graph, all nodes except the ends have degree 2, but the mean path length is of order n.

C Comparison of different tree balance statistics

Several measures exist that quantify the level of imbalance of phylogenetic trees (see [6] for an overview). Of these, the most commonly used are the Sackin index and the Colless index. The Sackin index sums the number of internal nodes d_i between each leaf i and the root of the tree,

$$I_S = \sum_{i=1}^{n} d_i, \tag{13}$$

while the Colless index compares the sizes of the right and left subtree and at each internal node (r_j and l_j respectively),

$$I_C = \frac{2}{(n-2)(n-1)} \sum_{j=1}^{n-1} |r_j - l_j|. \tag{14}$$

The normalizing constant above results from a maximally unbalanced tree, thus constraining I_C to the interval $[0, 1]$. It has been shown that these two measures of tree balance are highly correlated for trees generated by the Yule model and the uniform model [7]. In [8] Blum and François introduced a further shape statistic,

$$s = \sum_{j=1}^{n-1} \ln\left(N_j - 1\right). \tag{15}$$

It is possible to rewrite the Sackin index, such that

$$I_S = \sum_{i=1}^{n} d_i = \sum_{j=1}^{n-1} N_j, \tag{16}$$

which is the same as the s-Index without the logarithm up to a constant $n-1$. We calculated the different indices for trees generated under the ER, WS and BA network models for different population sizes and mean number of neighbors K. All indices are able to distinguish the WS and BA model from the ER model for large enough population sizes (see supporting text S2). Because of its wide-spread use and simple implementation, we have thus chosen the normalized Sackin index as the primary measure of tree imbalance in the main text.

D Testing the HIV tree using different tree imbalance statistics

We compare the tree imbalance of the phylogenetic tree from the Swiss HIV epidemic to trees generated under a birth-death process with density-dependent birth rates (SIR model with random mixing) using the measures mentioned in above. We are able to reject the birth-death process as a null model using the (normalized) Sackin index (and the Colless index, since these are strongly correlated) for the full tree as well as for the

three largest transmission clusters (see figure 7 in main text). It is not possible, however, to reject the birth-death process using the s-Index statistic. When comparing the joint distribution of the normalized Sackin index and the s-Index for trees generated by the SIR model, we show that only a small number of trees (437/10 000) generated by this process are rejected by the Sackin index test while not being rejected by the s-Index test (figure S3). This indicates that even though the HIV tree cannot be distinguished from an SIR model using the s-Index, it can reject the SIR model when using both both the s-Index as well as the Sackin index.

This argument is supported when looking at the sub-sampling behavior of the s-Index for the HIV tree as well as the transmission groups within. While we cannot reject the SIR model using the s-Index alone for the full HIV tree, the tree from a subsample of individuals is rejected by the s-Index, as are the transmission groups (see figure S4).

The values for N, M and R_0 in figure S3 were chosen to be realistic and conservative, however, it is possible that the SIR model with other parameters could no longer be rejected using tree imbalance statistics. Figure S6 shows the distribution of imbalance measures for trees generated by the SIR model under different parameterizations. The HIV tree rejects the SIR model for all of these parameter combinations. We thus consider this result stable.

E Path length

The control parameter in the Watts-Strogatz model is the rewiring probability p. When $p = 0$ no edges are rewired and the network is equal to a ring where each node is connected

to its K nearest neighbors. The degree distribution of such a network is,

$$p(k_i) = \begin{cases} 1 & \text{for } k_i = K, \\ 0 & \text{otherwise.} \end{cases} \tag{17}$$

As $p \to 1$ the network approaches a ER graph which has a Poisson degree distribution. The total number of links in the graph stays constant, such that,

$$\langle k \rangle_{p=0} = \langle k \rangle_{p=1} = \langle k \rangle_p = K \quad \forall p \in [0, 1].$$

Varying p affects two properties of the network: the clustering coefficient and the mean shortest path [4]. The strongest effect on path length is in the small p range, while the strongest effect on the clustering coefficient is in the large p range [4]. Figure S5 shows the dependence on the rewiring probability for a fixed transmissibility T and mean number of neighbors, compared to a random graph generated by the configuration model with degree sequence equal to that of th WS graph. The reduction in tree imbalance is strongest in the low p range, indicating that this effect is due to the reduction in mean path length, rather than reduction in the clustering coefficient or the variation in degree distribution.

References

1. Sackin M (1972) Good and bad phenograms. Systematic Zoology 21: 225-226.

2. Welch D, Bansal S, Hunter DR (2011) Statistical inference to advance network models in epidemiology. Epidemics 3: 38–45.

3. Barabási AL, Albert R (1999) Emergence of scaling in random networks. Science 286: 509–512.

4. Watts DJ, Strogatz SH (1998) Collective dynamics of 'small-world' networks. Nature 393: 440–442.

5. Erdős P, Rényi A (1959) On random graphs, i. Publicationes Mathematicae Debrecen 6: 290–297.

6. Kirkpatrick M, Slatkin M (1993) Searching for evolutionary patterns in the shape of a phylogenetic tree. Evolution 47: 1171–1181.

7. Blum MGB, FranÃ§ois O, Janson S (2006) The mean, variance and limiting distribution of two statistics sensitive to phylogenetic tree balance. The Annals of Applied Probability 16: 2195–2214.

8. Blum MGB, François O (2006) Which random processes describe the tree of life? a large-scale study of phylogenetic tree imbalance. Systematic Biology 55: 685-691.
