# Supplementary material for: Inferring Epidemic Contact Structure from Phylogenetic Trees
Source: PLoS Comput Biol. 2012 Mar 8;8(3):e1002413. doi: 10.1371/journal.pcbi.1002413 (PMC3297558; doi:10.1371/journal.pcbi.1002413)
Supplement: Text S2 — Detailed analysis of population size N and mean number of neighbors k on tree imbalance. (PDF) [file pcbi.1002413.s009.pdf]

Inferring Epidemic Contact Structure from Phylogenetic Trees: Supporting Text S2

Comparison of different imbalance statistics

Gabriel E Leventhal[1,*], Roger Kouyos[2], Tanja Stadler[1], Viktor von Wyl[3], Sabine Yerly[4], Jürg Böni[5], Cristina Cellerai[6], Thomas Klimkait[7], Huldrych F. Günthard[3], and Sebastian Bonhoeffer[1]

[1]Institute of Integrative Biology, ETH Zurich, Zurich, Switzerland

[2]Department of Ecology and Evolutionary Biology, Princeton University, Princeton, New Jersey, United States of America

[3]Division of Infectious Diseases and Hospital Epidemiology, University Hospital Zurich, Zurich, Switzerland

[4]Laboratory of Virology and AIDS Center, Geneva University Hospital, Geneva, Switzerland

[5]Swiss National Center for Retroviruses, Institute of Medical Virology, University of Zurich, Zurich, Switzerland

[6]Service of Immunology and Allergy, Lausanne University Hospital, Lausanne, Switzerland

[7]Institute of Medical Microbiology, Department Biomedicine, University of Basel, Basel, Switzerland

[*]E-mail: gabriel.leventhal@env.ethz.ch

January 25, 2012

The following plots show a detailed comparison of different imbalance statistics of trees genertated by SIR epidemics with contact networks from the three different models (ER, WS and BA). K is the mean number of neighbors in the network and N the total population size. For all plots, the WS network has rewiring probability $p = 0.01$. The solid curves show the median over 2 000 independent networks for each model. The light and dark shaded areas are the 50% and 95% confidence intervals.

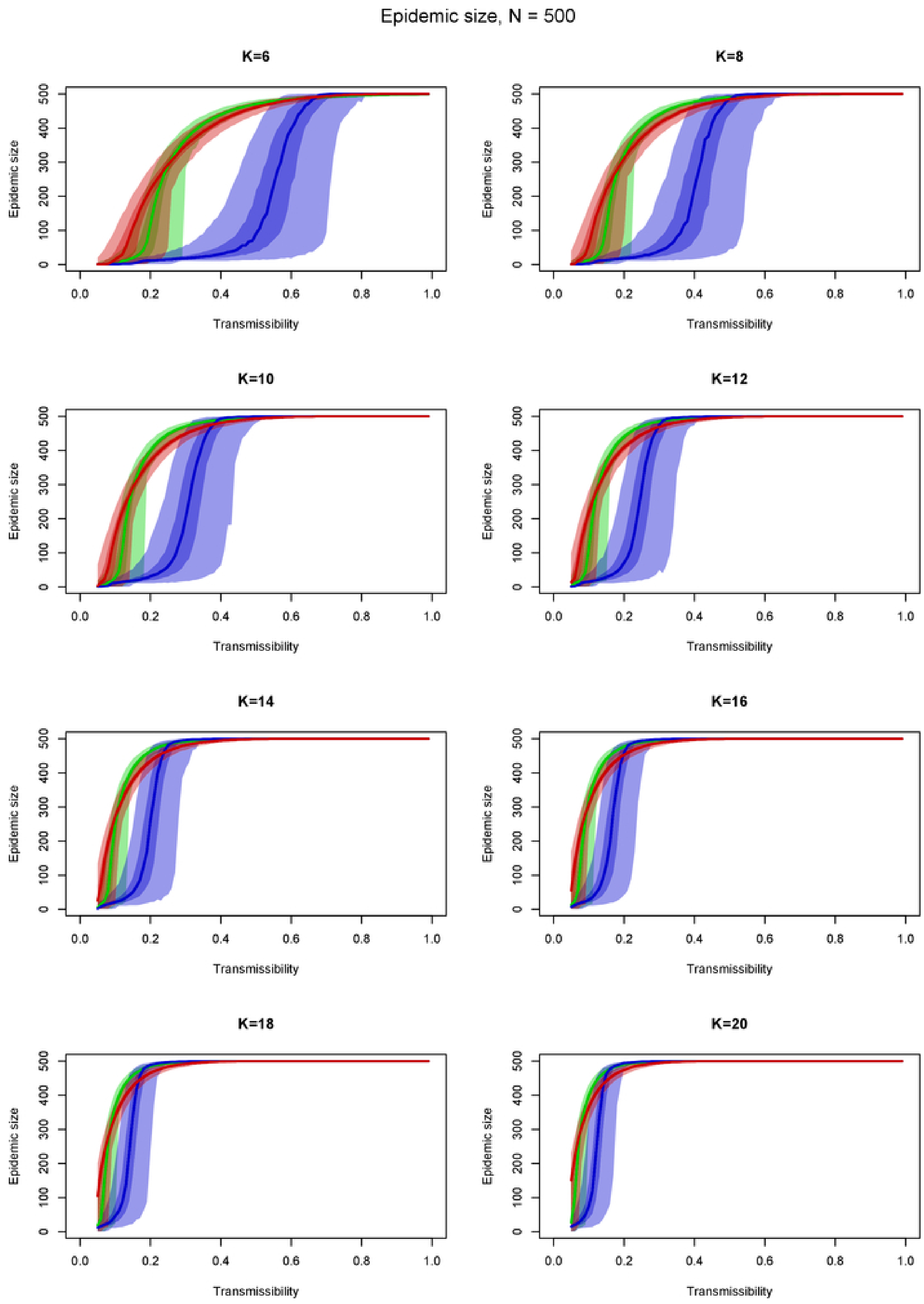
Epidemic size, N = 500
K=6
K=8
K=10
K=12
K=14
K=16
K=18
K=20
Epidemic size
0
100
200
300
400
500
Transmissibility
0.0
0.2
0.4
0.6
0.8
1.0

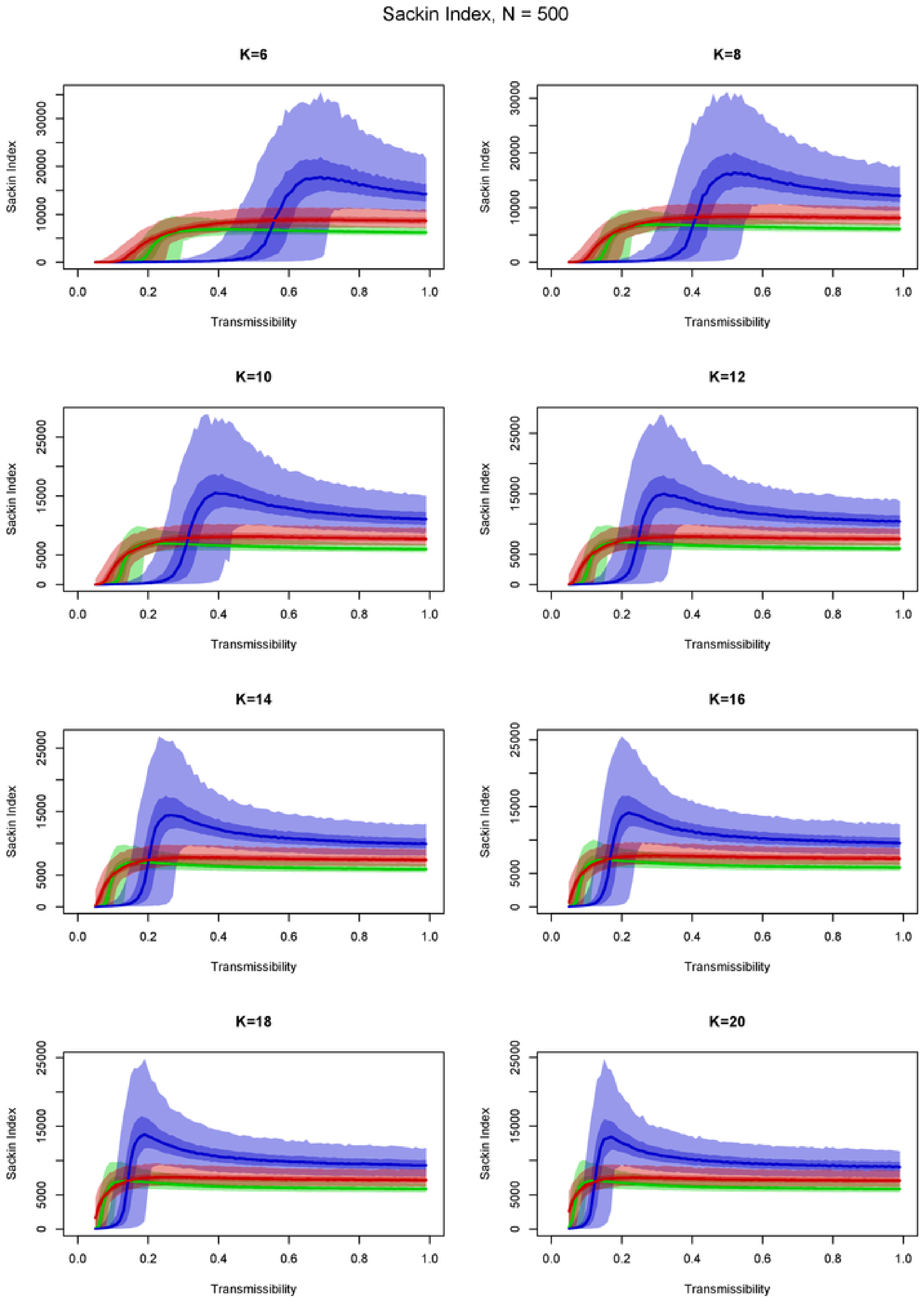
Sackin Index, N = 500
K=6
K=8
K=10
K=12
K=14
K=16
K=18
K=20
Sackin Index
Transmissibility
0.0
0.2
0.4
0.6
0.8
1.0
0
5000
10000
15000
20000
25000
30000

Colless Index, N = 500

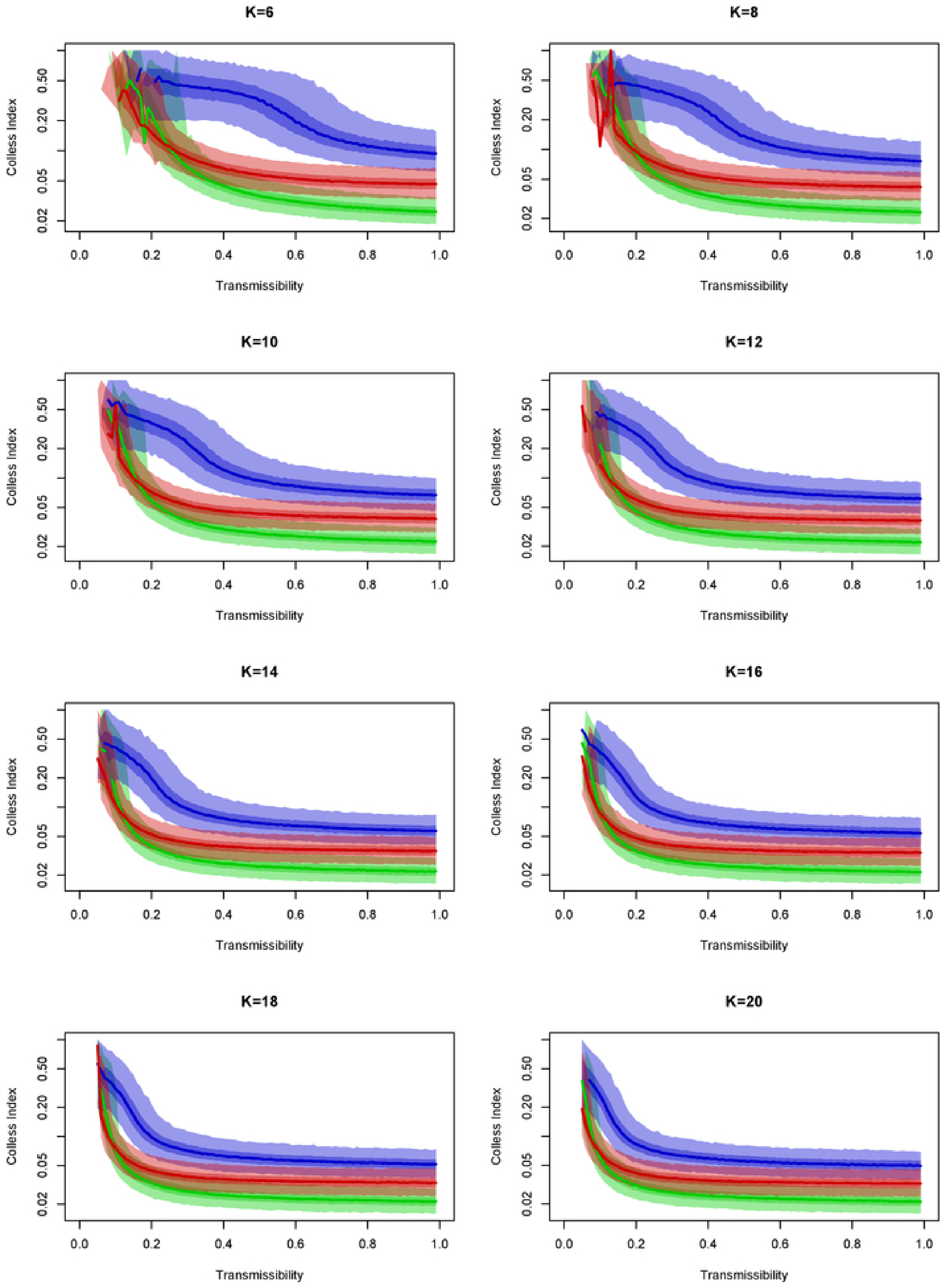

Normalized Sackin Index, N = 500

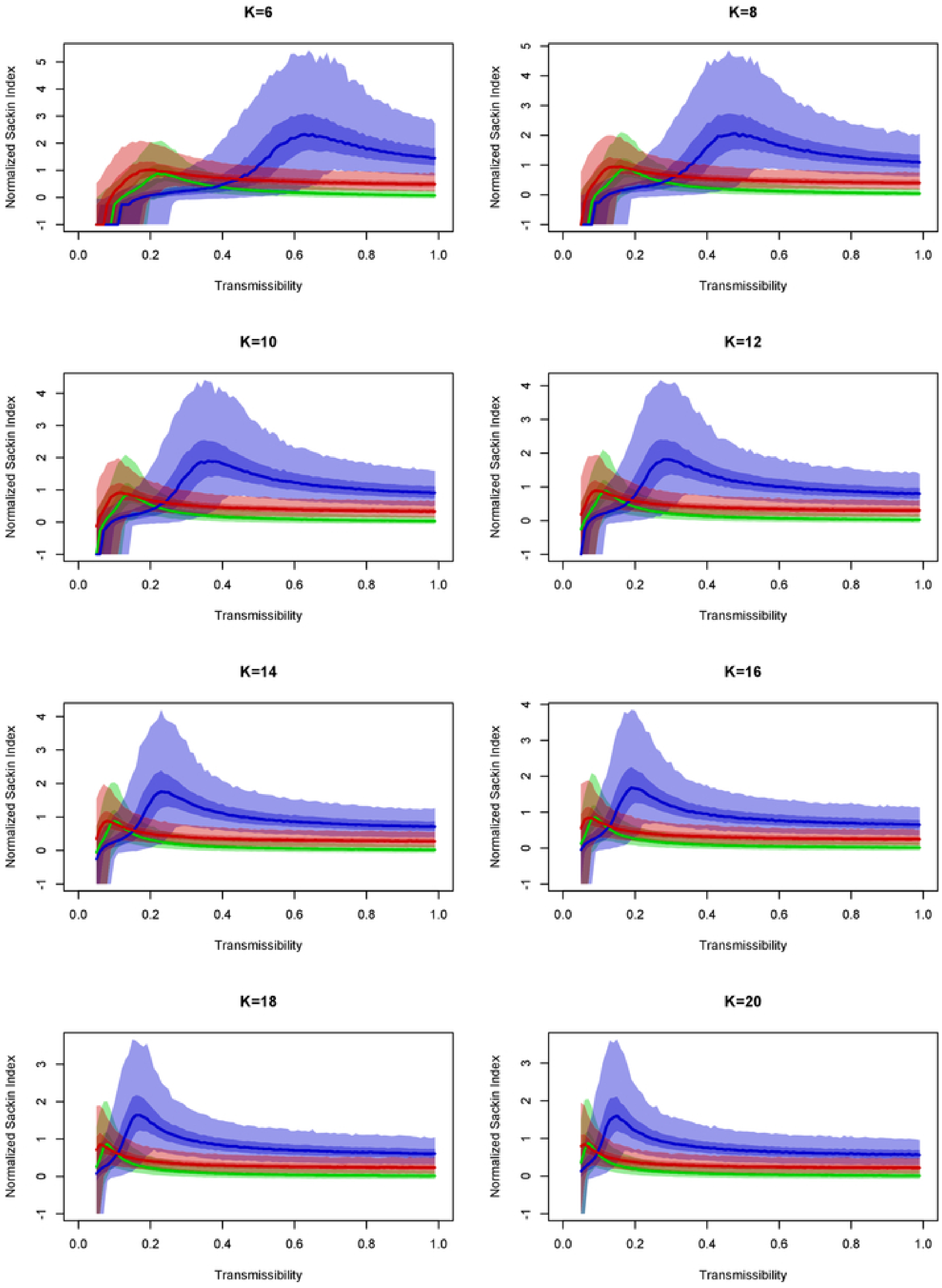

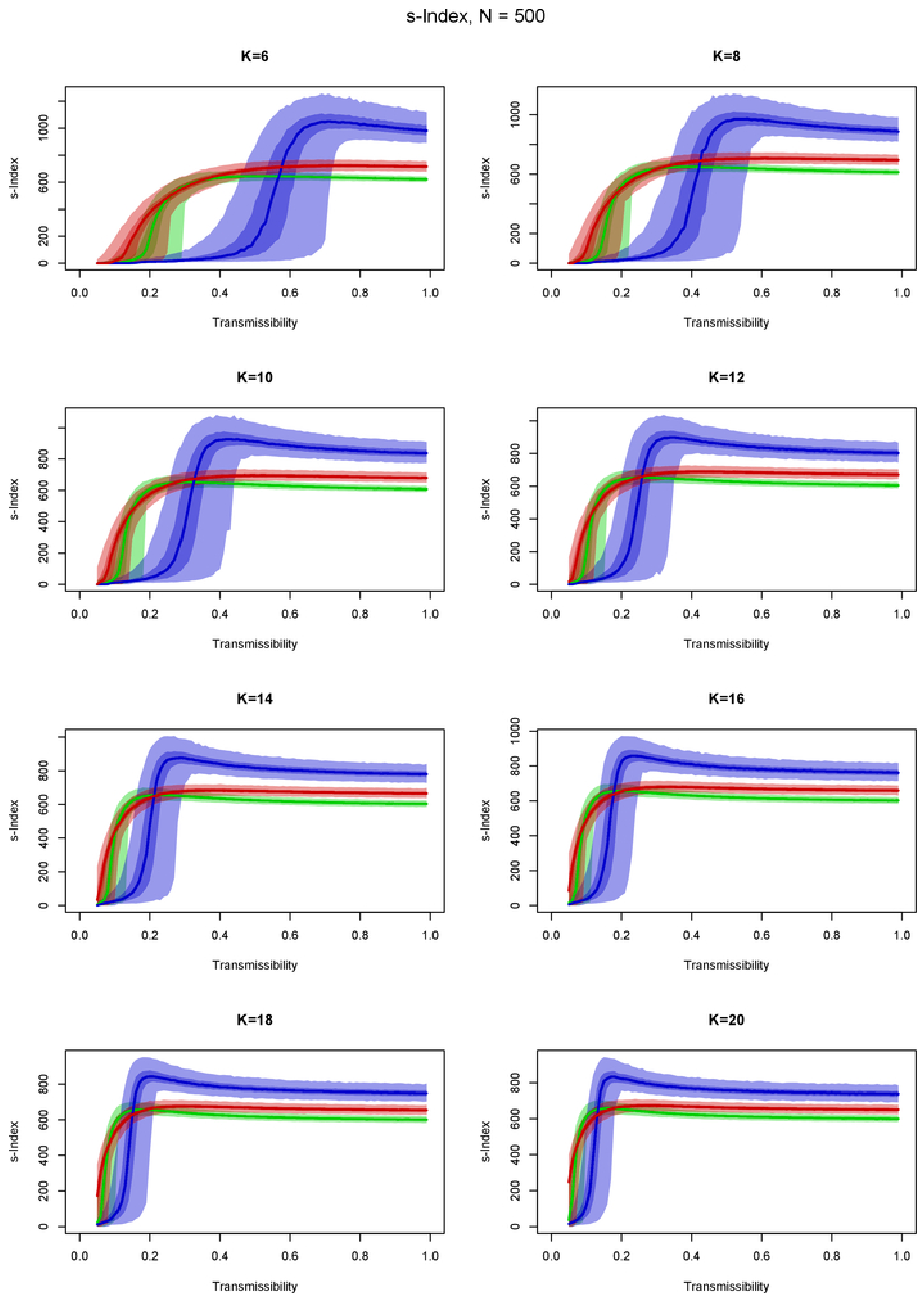
s-Index, N = 500
K=6
K=8
K=10
K=12
K=14
K=16
K=18
K=20
s-Index
Transmissibility
0.0
0.2
0.4
0.6
0.8
1.0
0
200
400
600
800
1000

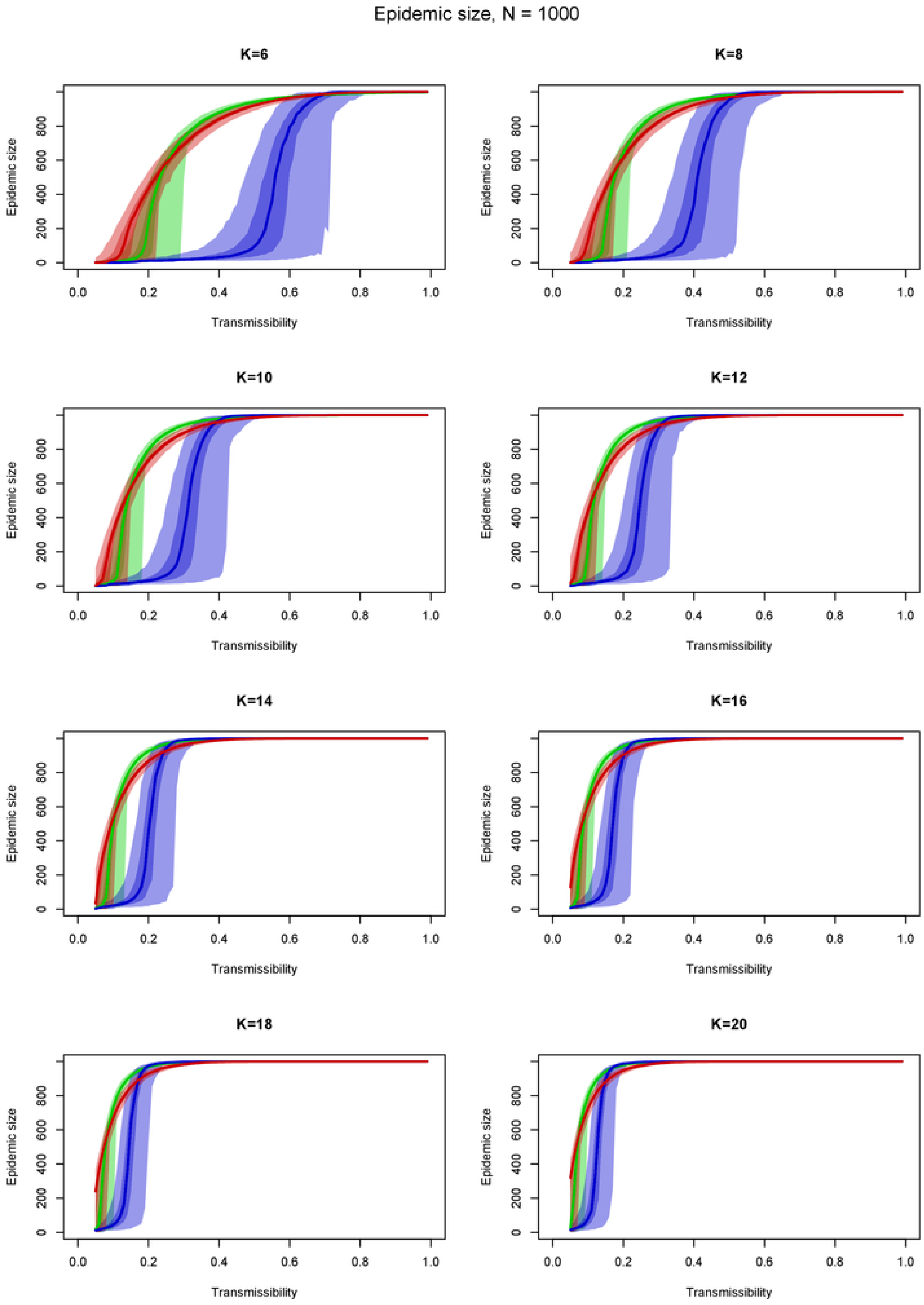
Epidemic size, N = 1000
K=6
K=8
K=10
K=12
K=14
K=16
K=18
K=20
Epidemic size
0
200
400
600
800
Transmissibility
0.0
0.2
0.4
0.6
0.8
1.0

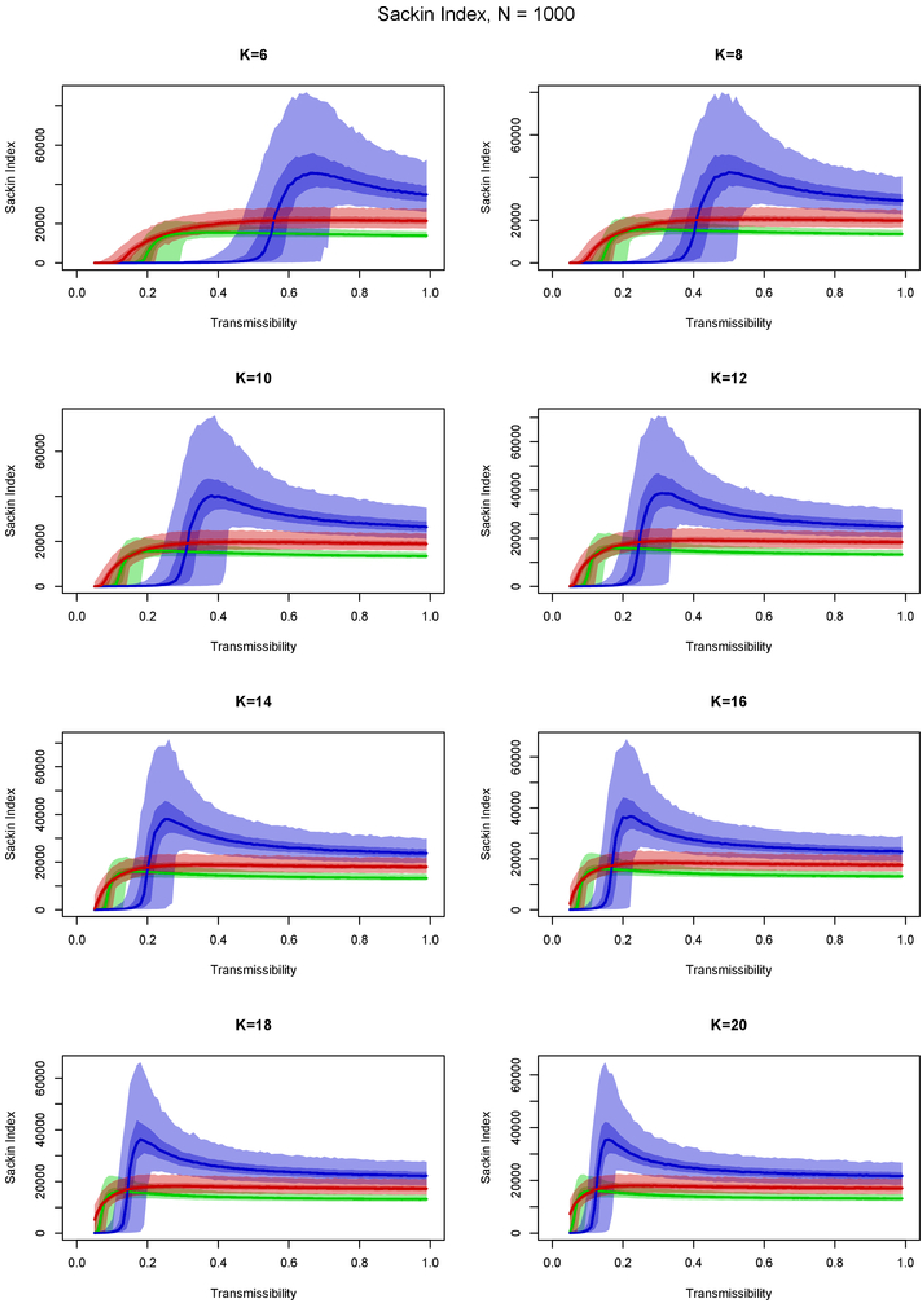
Sackin Index, N = 1000
K=6
K=8
K=10
K=12
K=14
K=16
K=18
K=20
Sackin Index
Transmissibility
0
20000
40000
60000
0.0
0.2
0.4
0.6
0.8
1.0

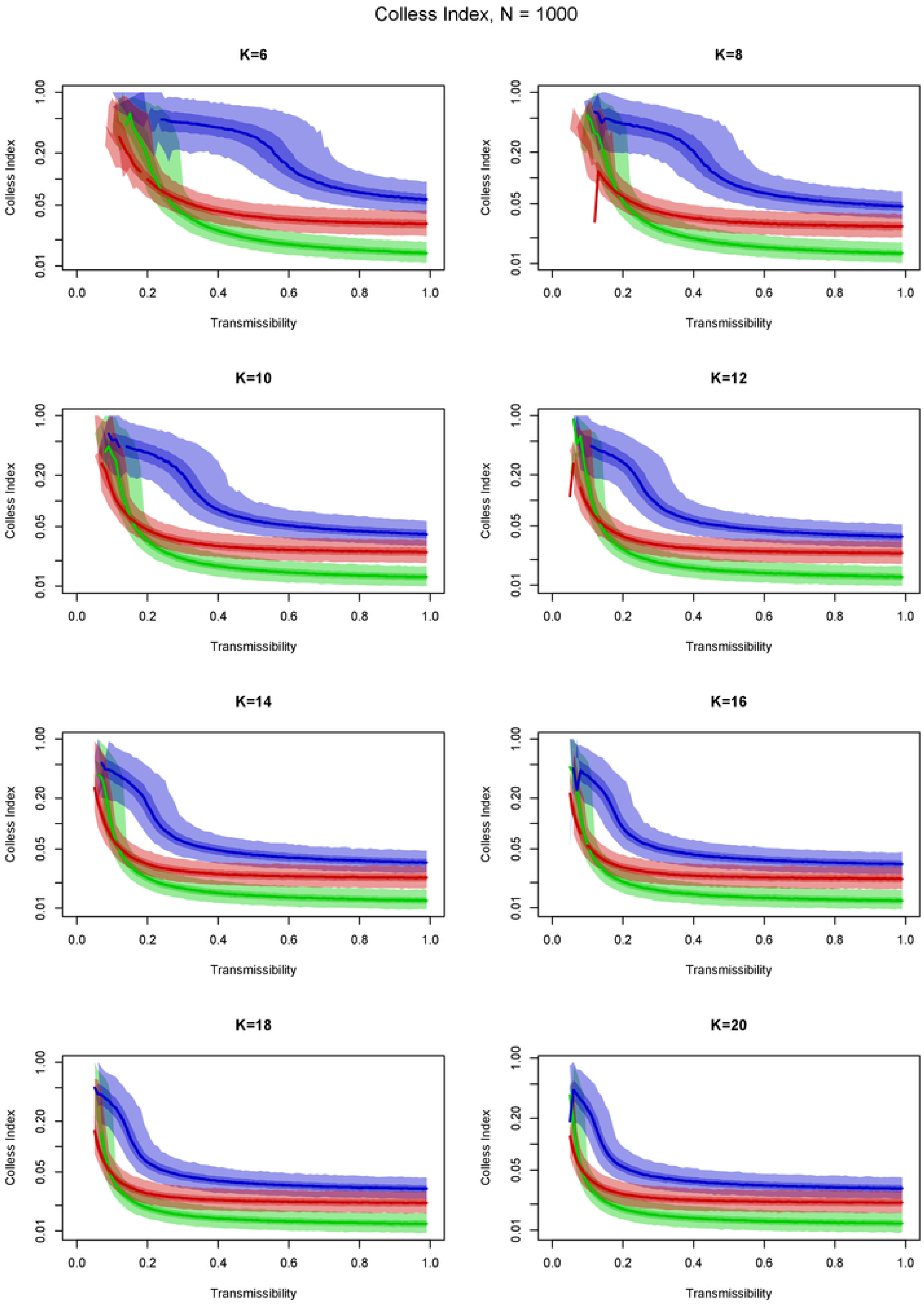
Colless Index, N = 1000
K=6
K=8
K=10
K=12
K=14
K=16
K=18
K=20
Colless Index
0.01
0.05
0.20
1.00
Transmissibility
0.0
0.2
0.4
0.6
0.8
1.0

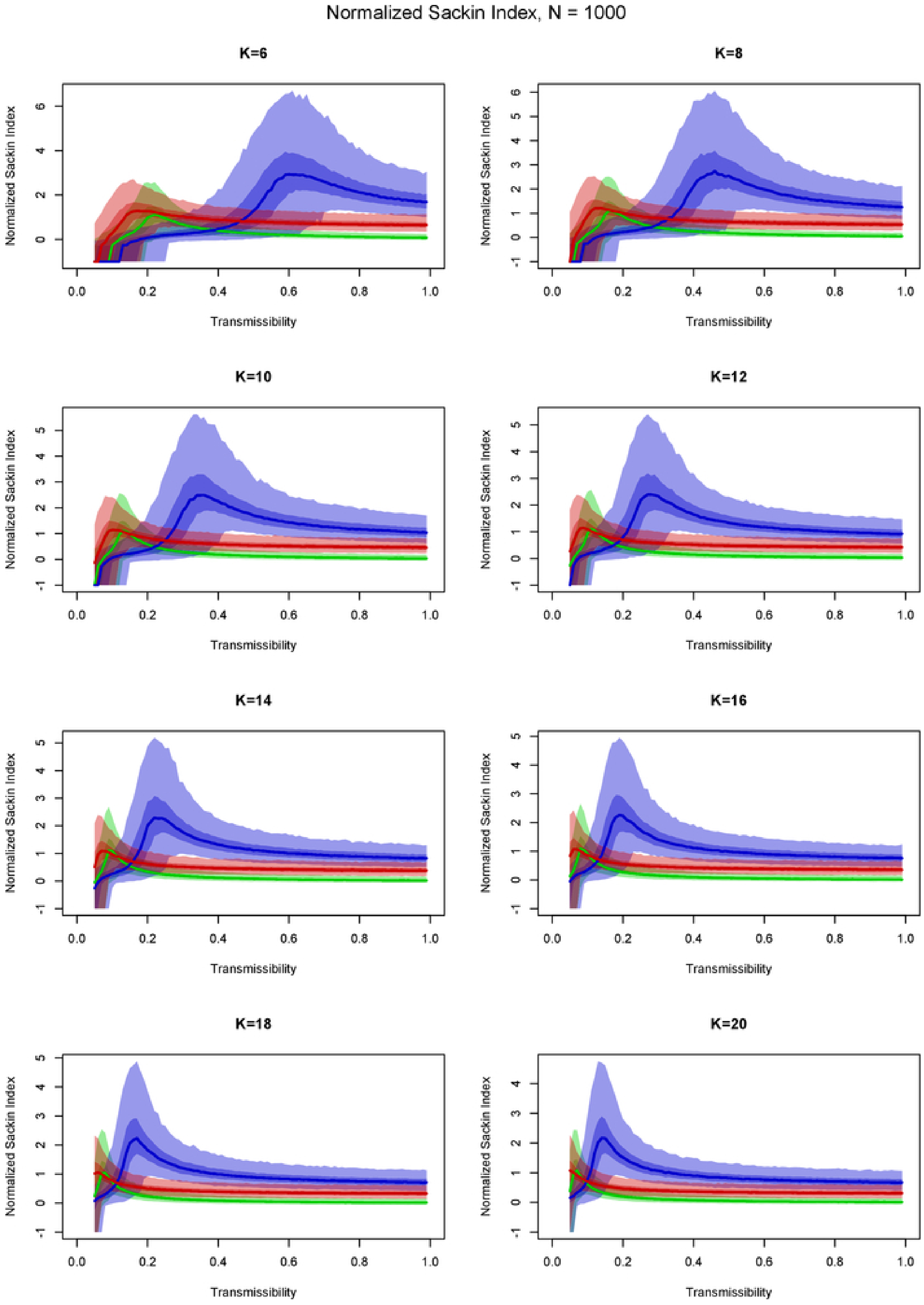
Normalized Sackin Index, N = 1000
K=6
K=8
K=10
K=12
K=14
K=16
K=18
K=20
Normalized Sackin Index
Transmissibility

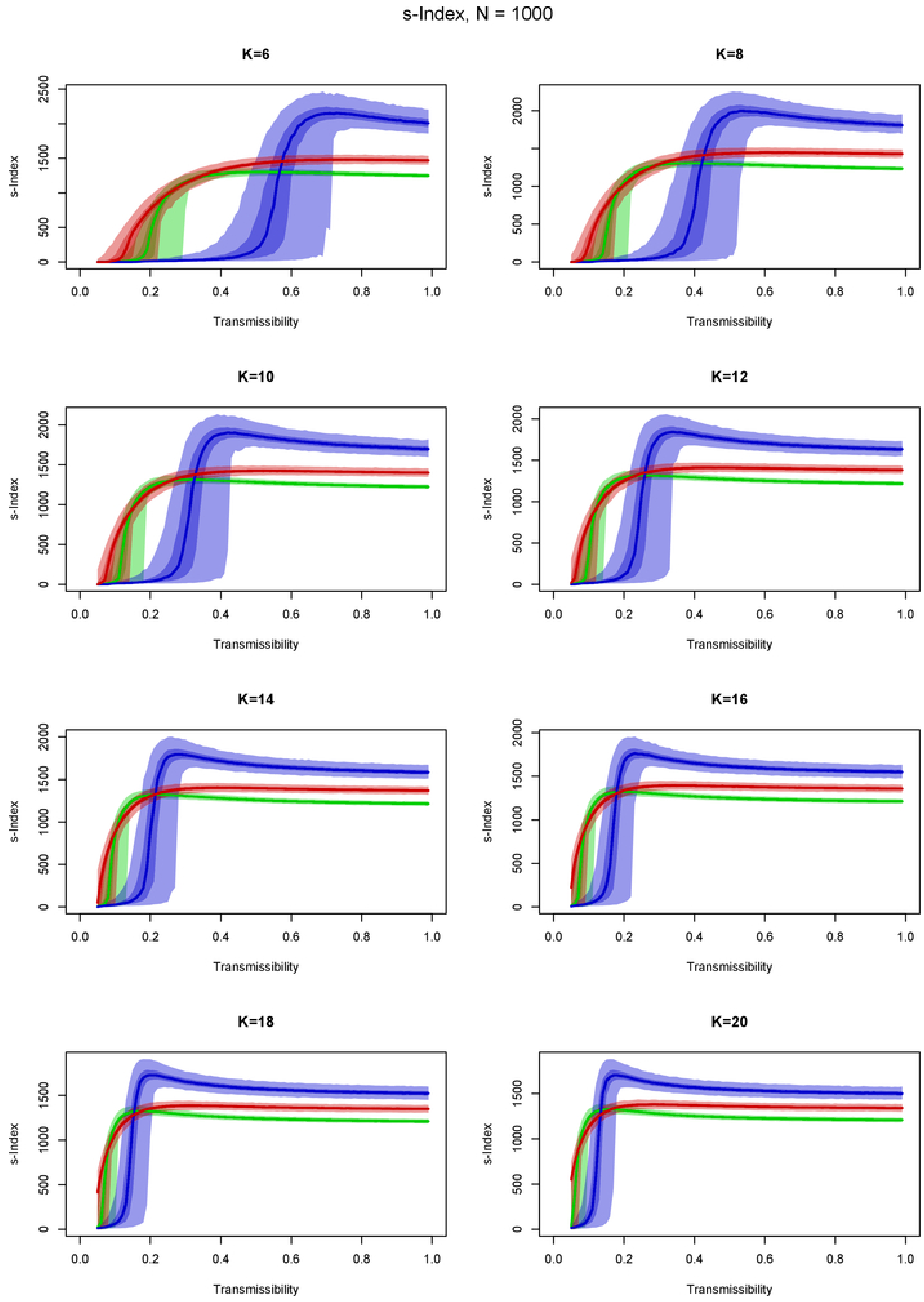
s-Index, N = 1000
K=6
K=8
K=10
K=12
K=14
K=16
K=18
K=20
s-Index
Transmissibility
0.0
0.2
0.4
0.6
0.8
1.0
0
500
1000
1500
2000
2500

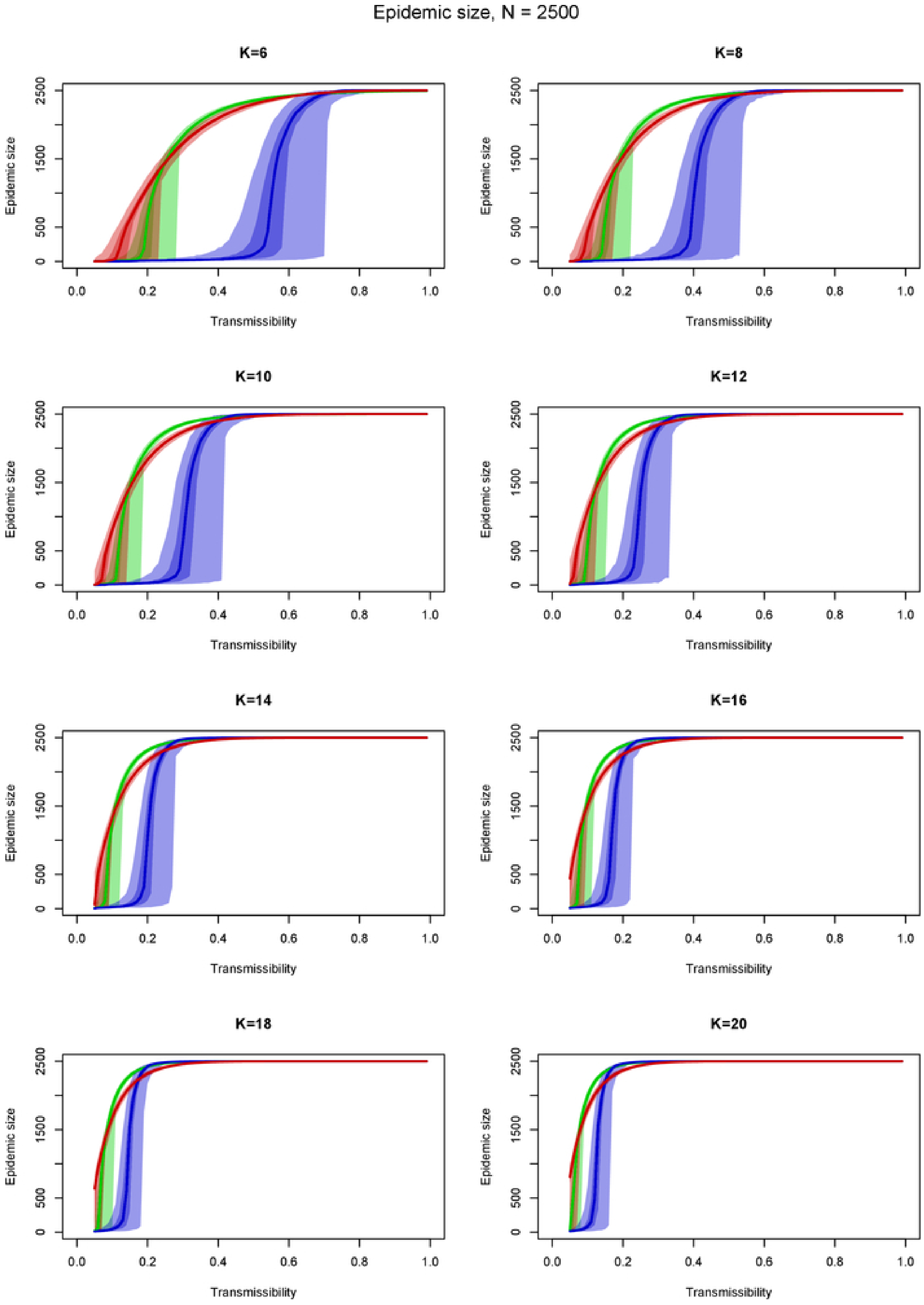
Epidemic size, N = 2500
K=6
K=8
K=10
K=12
K=14
K=16
K=18
K=20
Epidemic size
Transmissibility
0
500
1500
2500
0.0
0.2
0.4
0.6
0.8
1.0

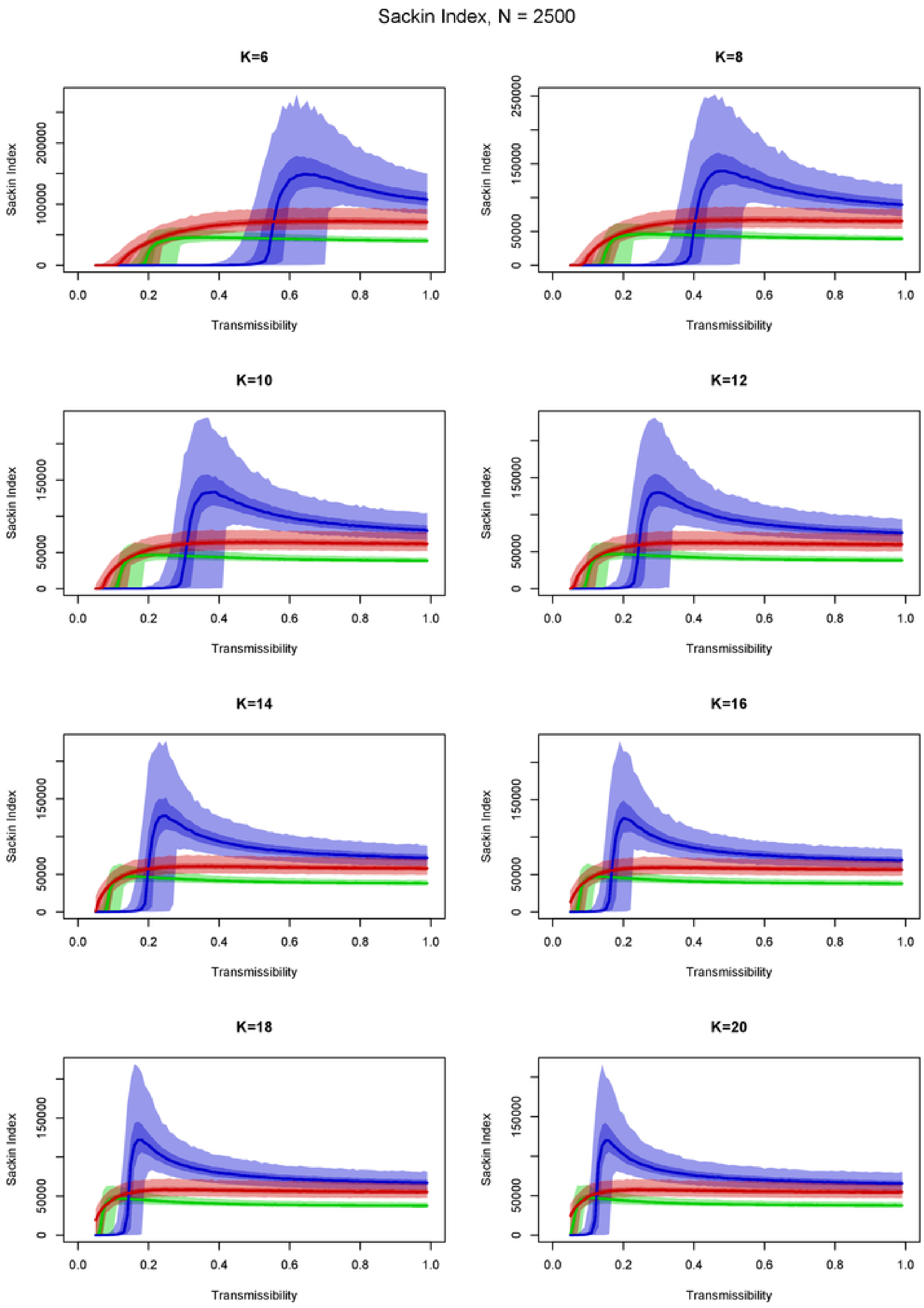
Sackin Index, N = 2500
K=6
K=8
K=10
K=12
K=14
K=16
K=18
K=20
Sackin Index
Transmissibility
0
50000
100000
150000
200000
250000
0.0
0.2
0.4
0.6
0.8
1.0

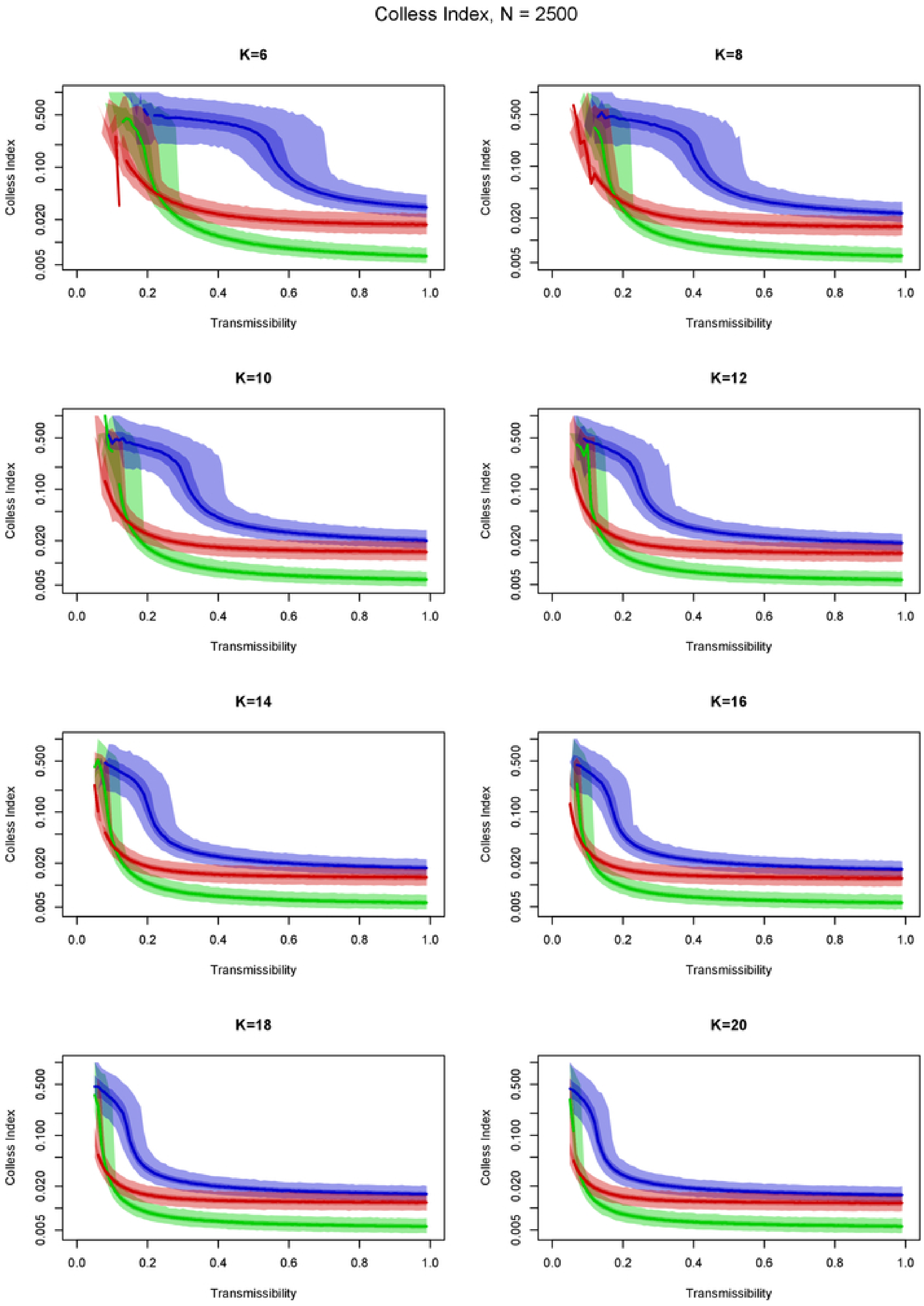
Colless Index, N = 2500
K=6
K=8
K=10
K=12
K=14
K=16
K=18
K=20
Colless Index
0.005
0.020
0.100
0.500
Transmissibility
0.0
0.2
0.4
0.6
0.8
1.0

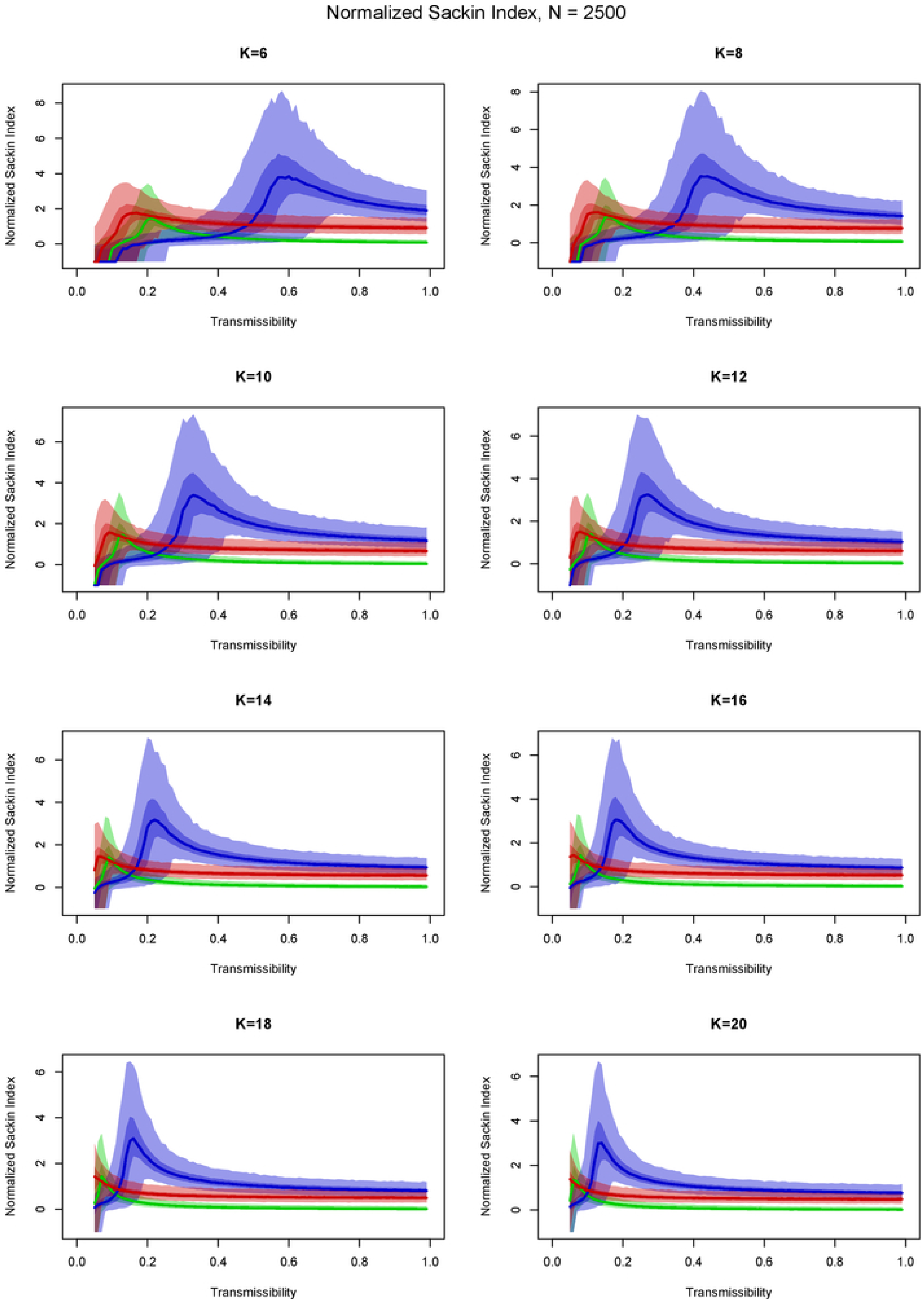
Normalized Sackin Index, N = 2500
K=6
K=8
K=10
K=12
K=14
K=16
K=18
K=20
Normalized Sackin Index
Transmissibility
0.0
0.2
0.4
0.6
0.8
1.0
0
2
4
6
8

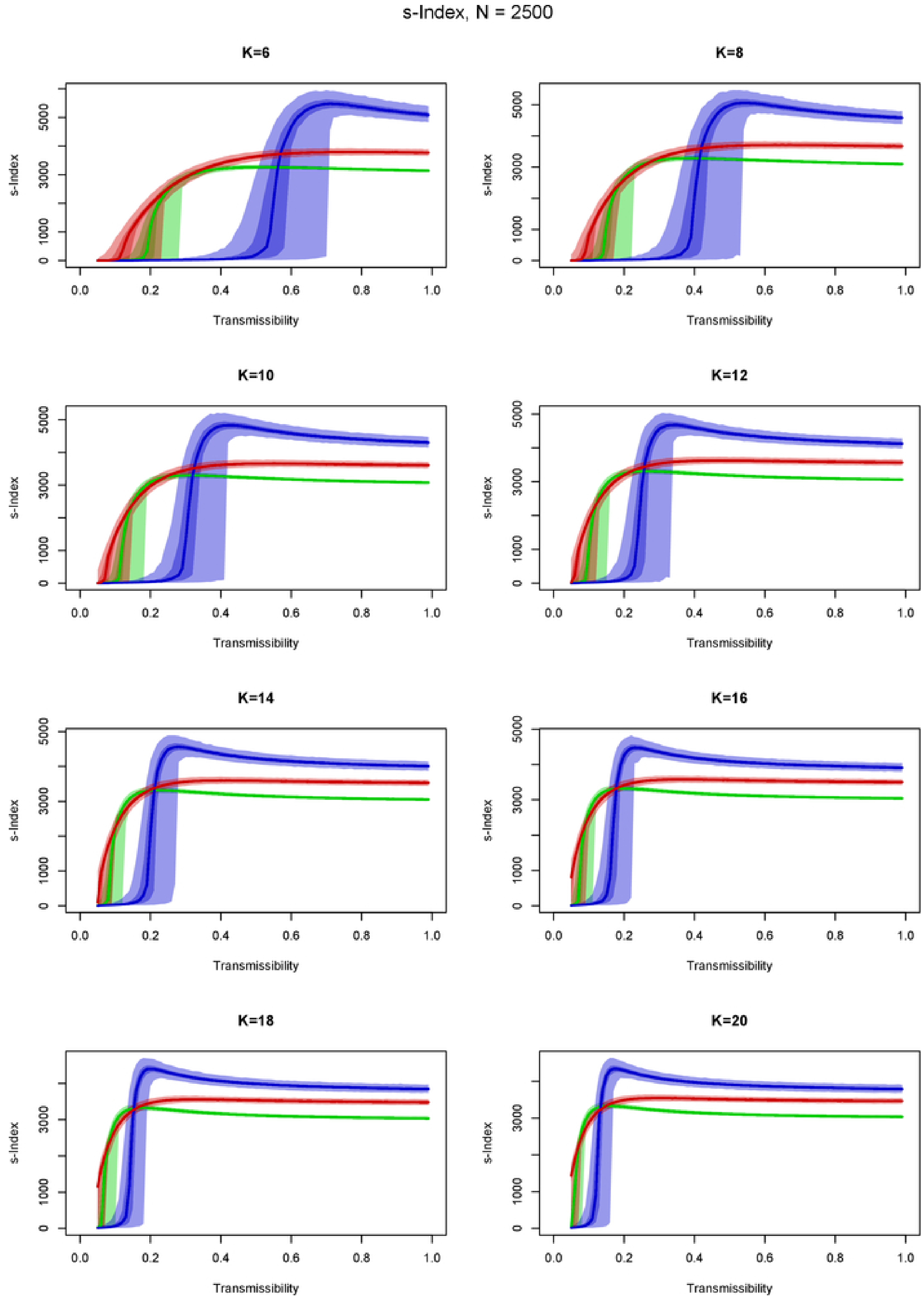
s-Index, N = 2500
K=6
K=8
K=10
K=12
K=14
K=16
K=18
K=20
s-Index
Transmissibility
0.0
0.2
0.4
0.6
0.8
1.0
0
1000
3000
5000

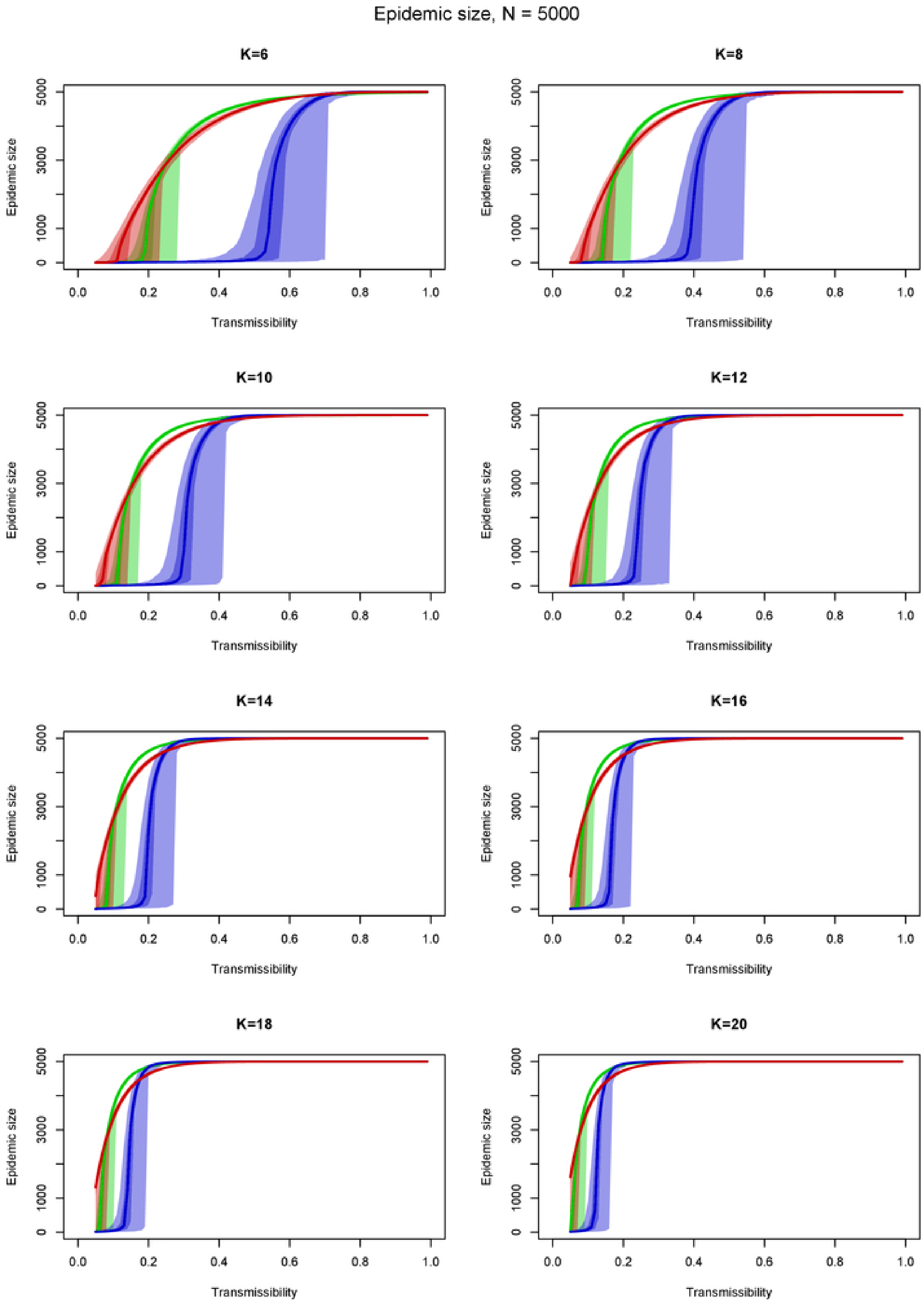
Epidemic size, N = 5000
K=6
K=8
K=10
K=12
K=14
K=16
K=18
K=20
Epidemic size
0
1000
3000
5000
Transmissibility
0.0
0.2
0.4
0.6
0.8
1.0

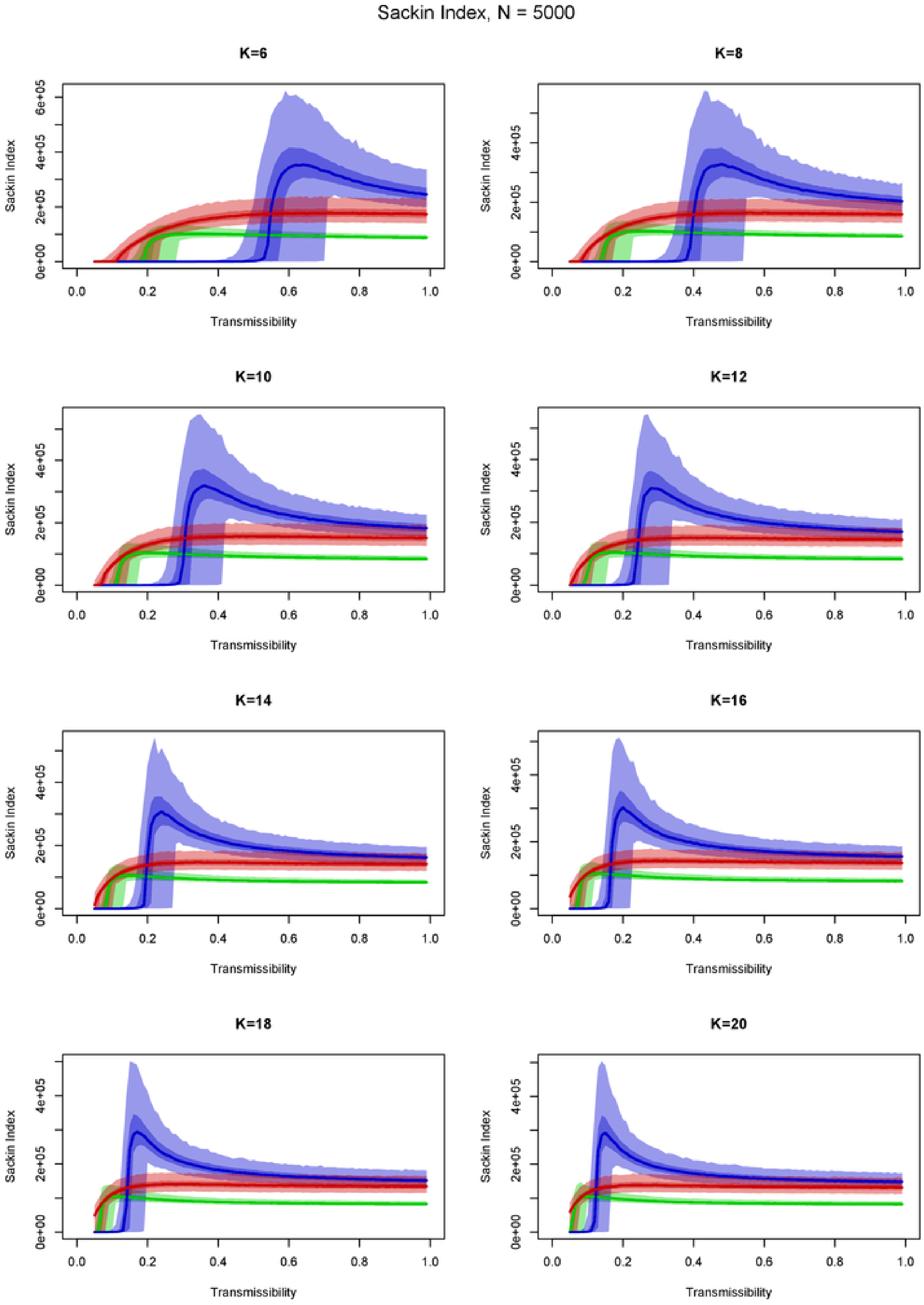
Sackin Index, N = 5000
K=6
K=8
K=10
K=12
K=14
K=16
K=18
K=20
Sackin Index
Transmissibility
0e+00
2e+05
4e+05
6e+05
0.0
0.2
0.4
0.6
0.8
1.0

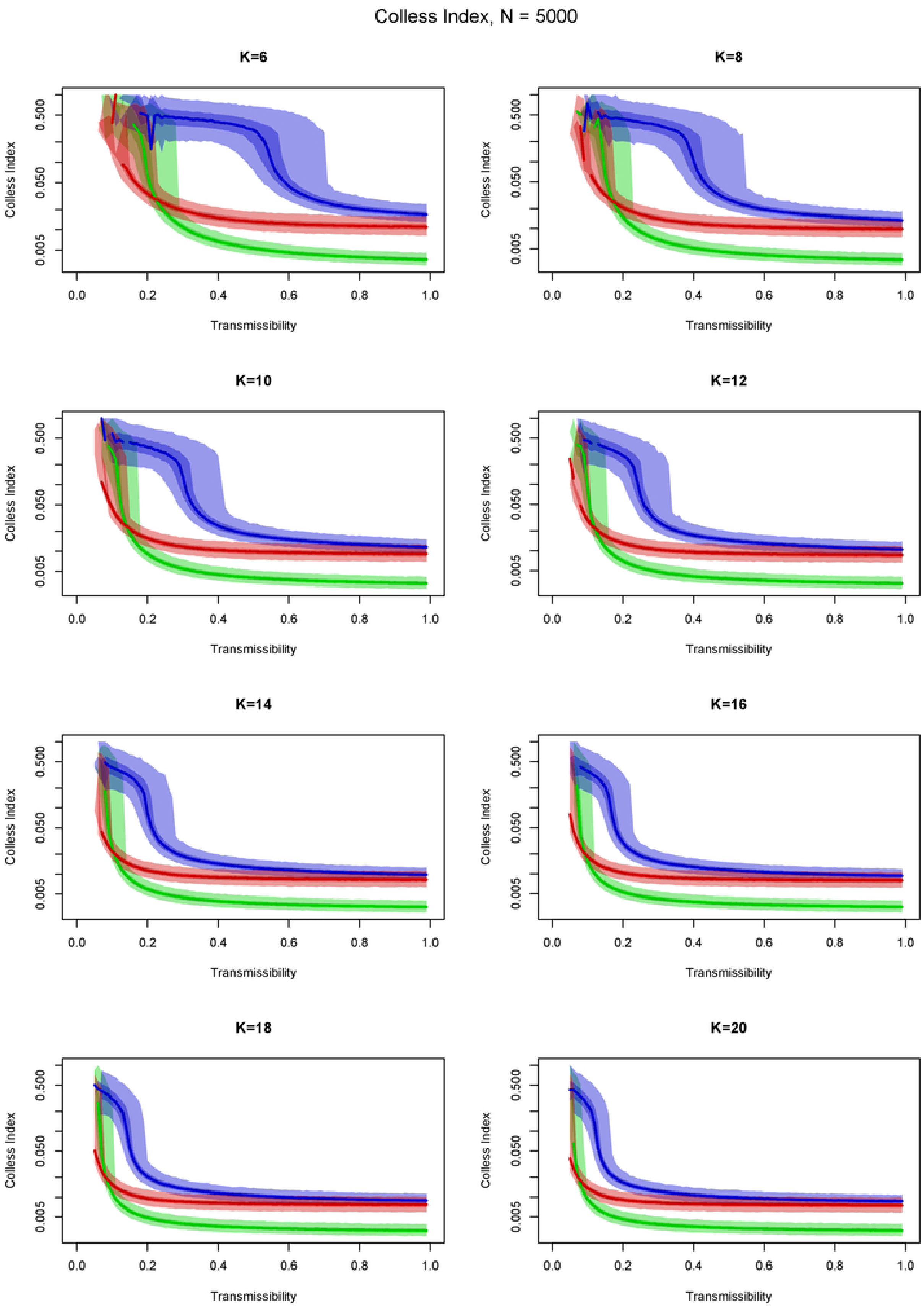
Colless Index, N = 5000
K=6
K=8
K=10
K=12
K=14
K=16
K=18
K=20
Colless Index
0.005
0.050
0.500
Transmissibility
0.0
0.2
0.4
0.6
0.8
1.0

Normalized Sackin Index, N = 5000

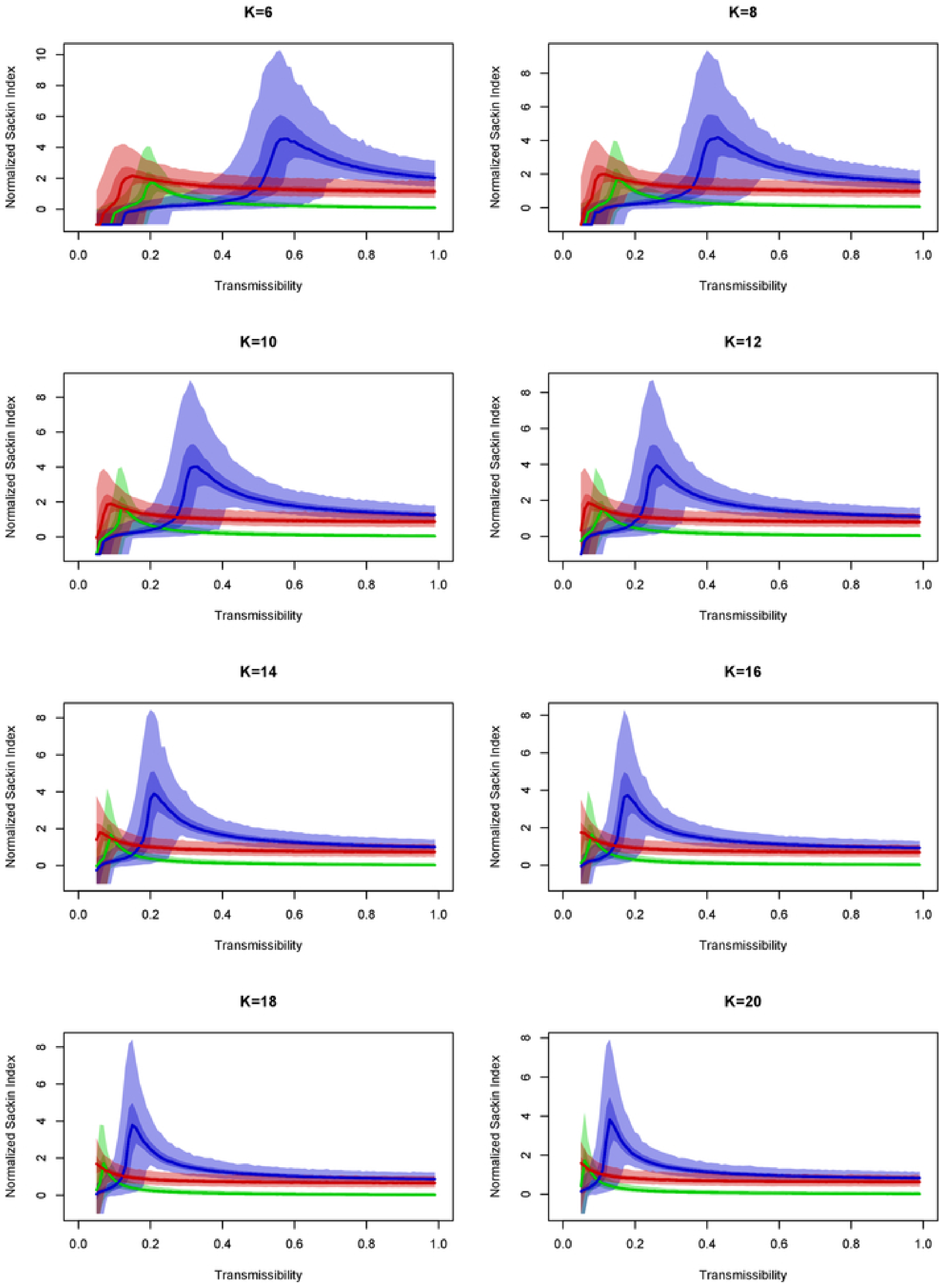

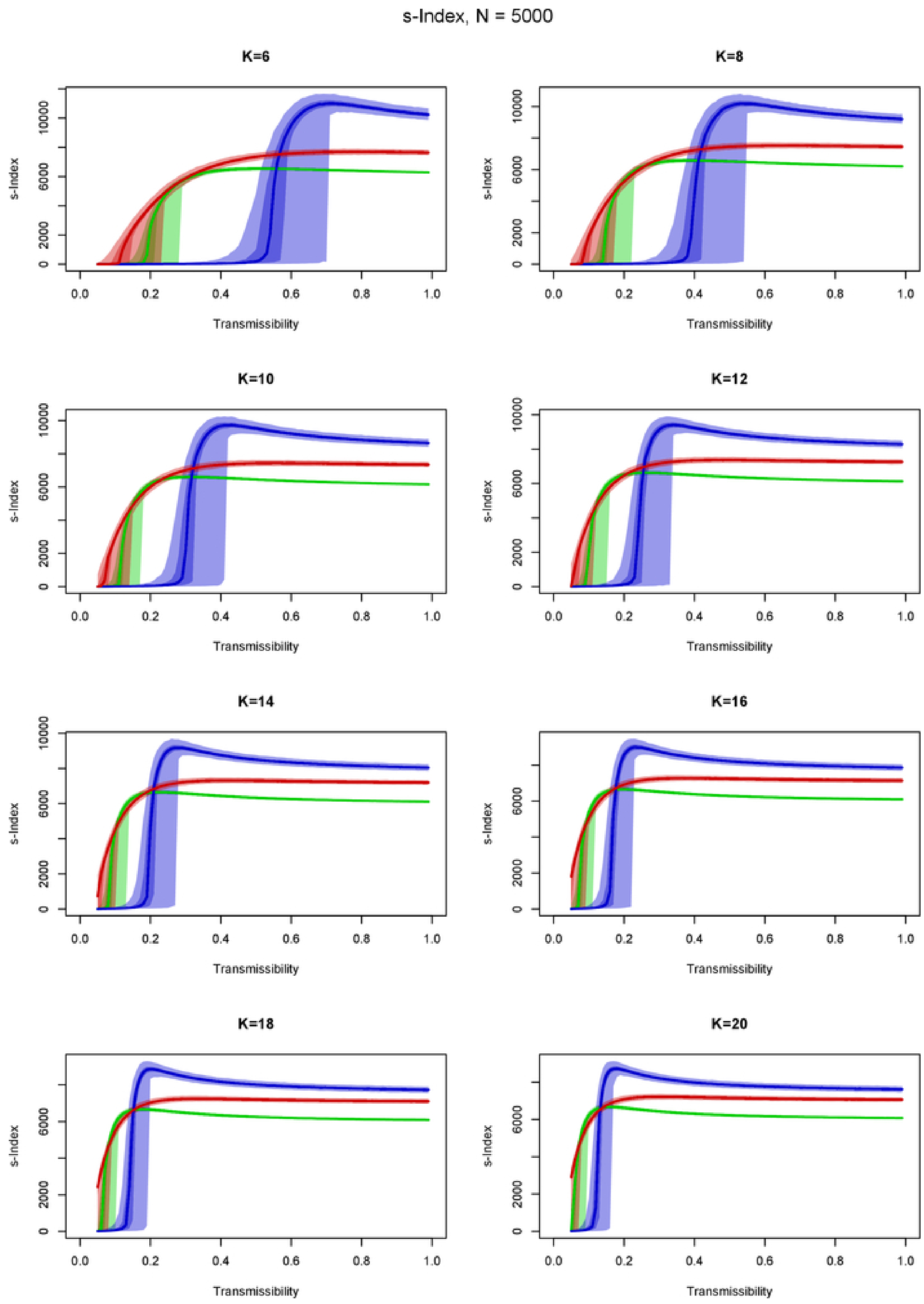
s-Index, N = 5000
K=6
K=8
K=10
K=12
K=14
K=16
K=18
K=20
s-Index
Transmissibility
0.0
0.2
0.4
0.6
0.8
1.0
0
2000
6000
10000

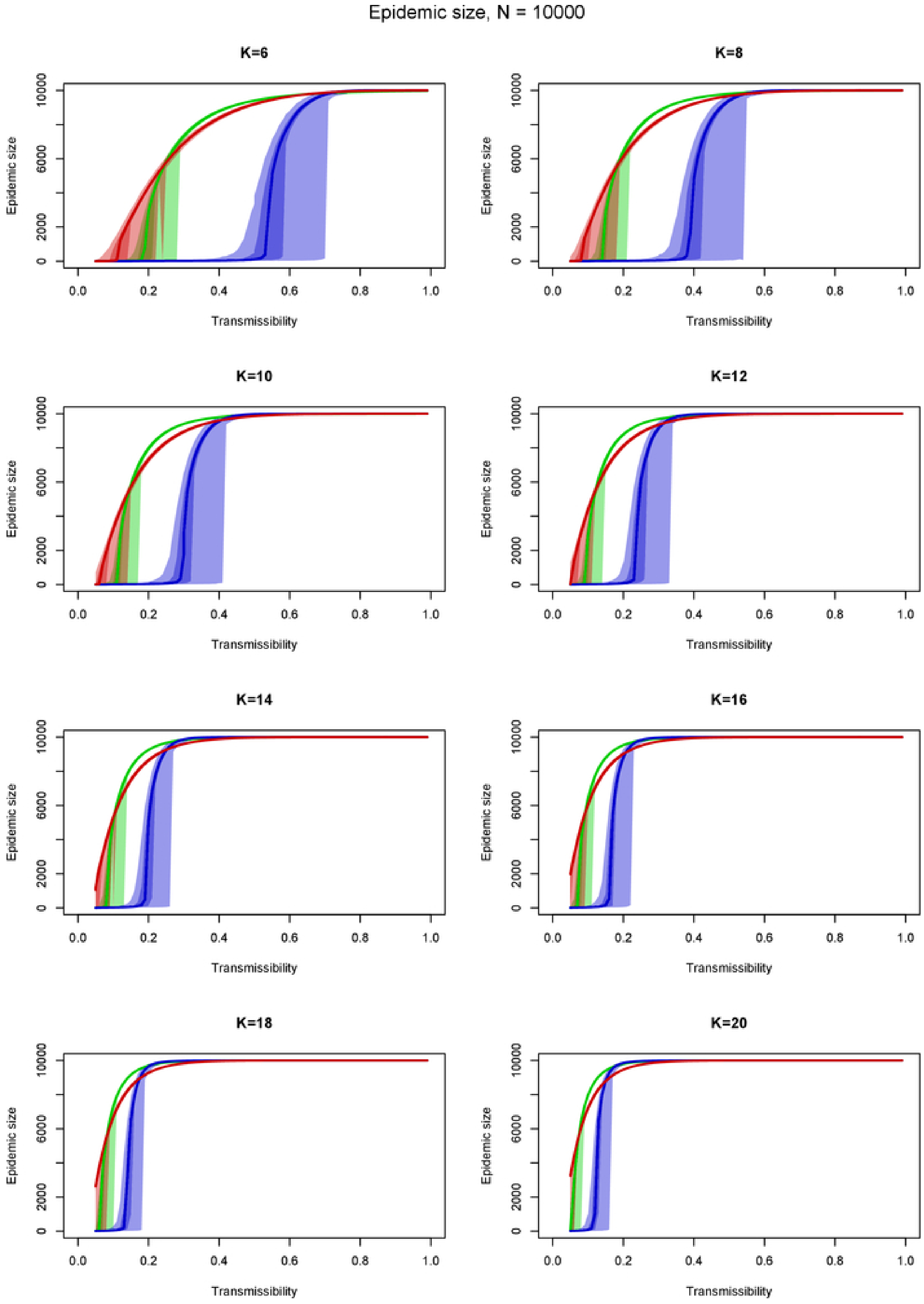

Epidemic size, N = 10000
K=6
K=8
K=10
K=12
K=14
K=16
K=18
K=20
Epidemic size
Transmissibility
0
2000
6000
10000
0.0
0.2
0.4
0.6
0.8
1.0

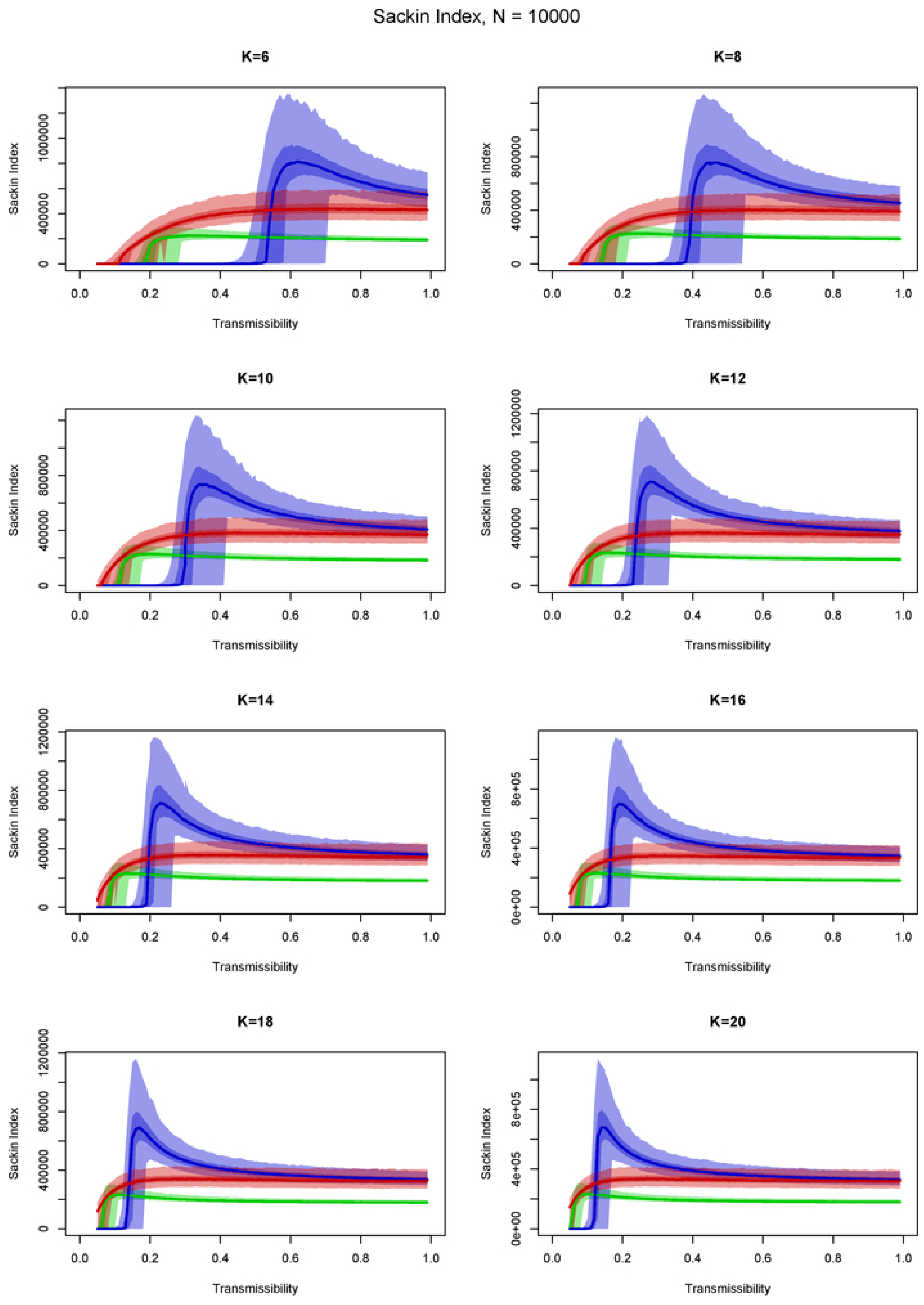
Sackin Index, N = 10000
K=6
K=8
K=10
K=12
K=14
K=16
K=18
K=20
Sackin Index
Transmissibility

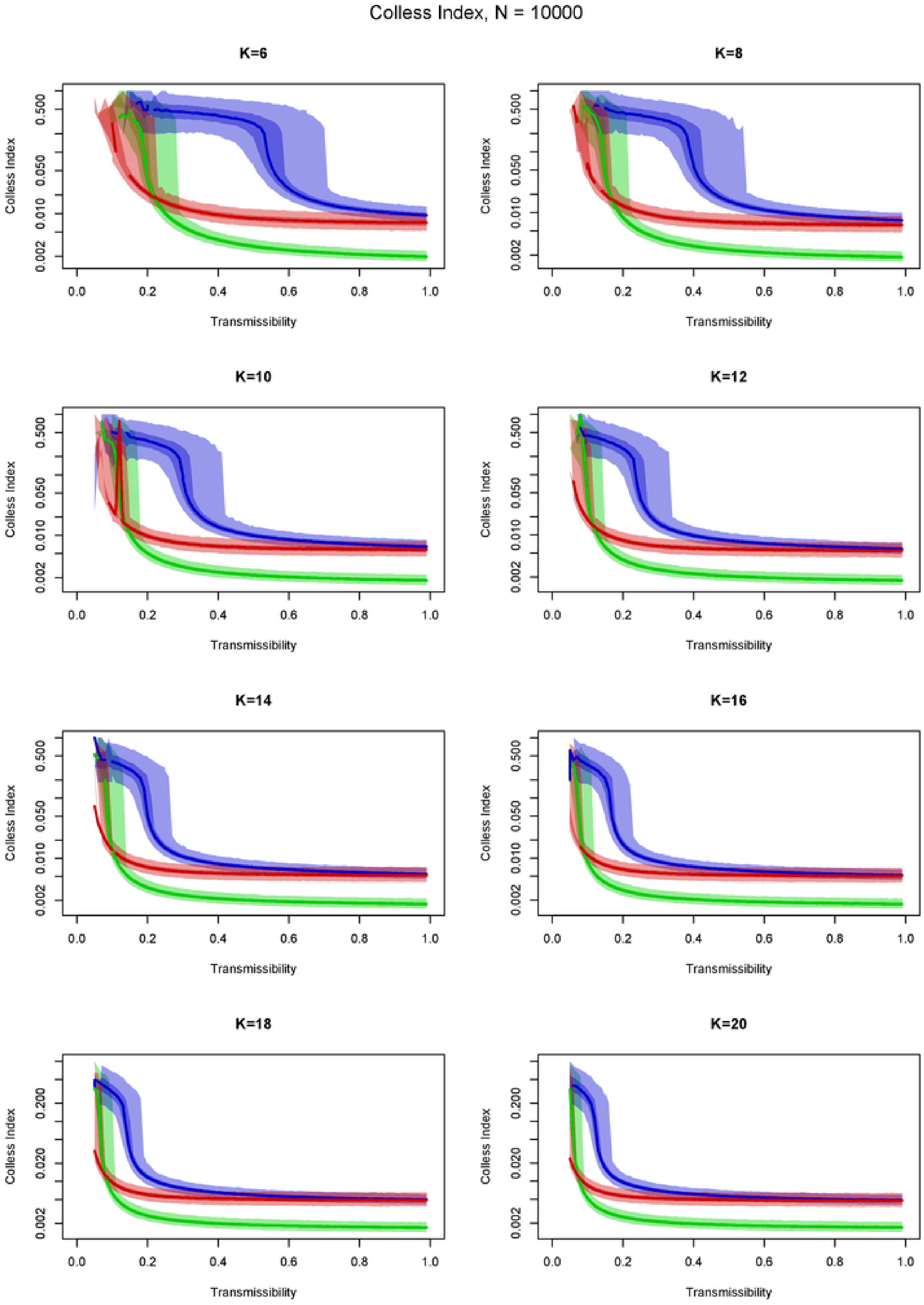
Colless Index, N = 10000
K=6
K=8
K=10
K=12
K=14
K=16
K=18
K=20
Colless Index
Transmissibility
0.0
0.2
0.4
0.6
0.8
1.0
0.002
0.010
0.050
0.500
0.020
0.200

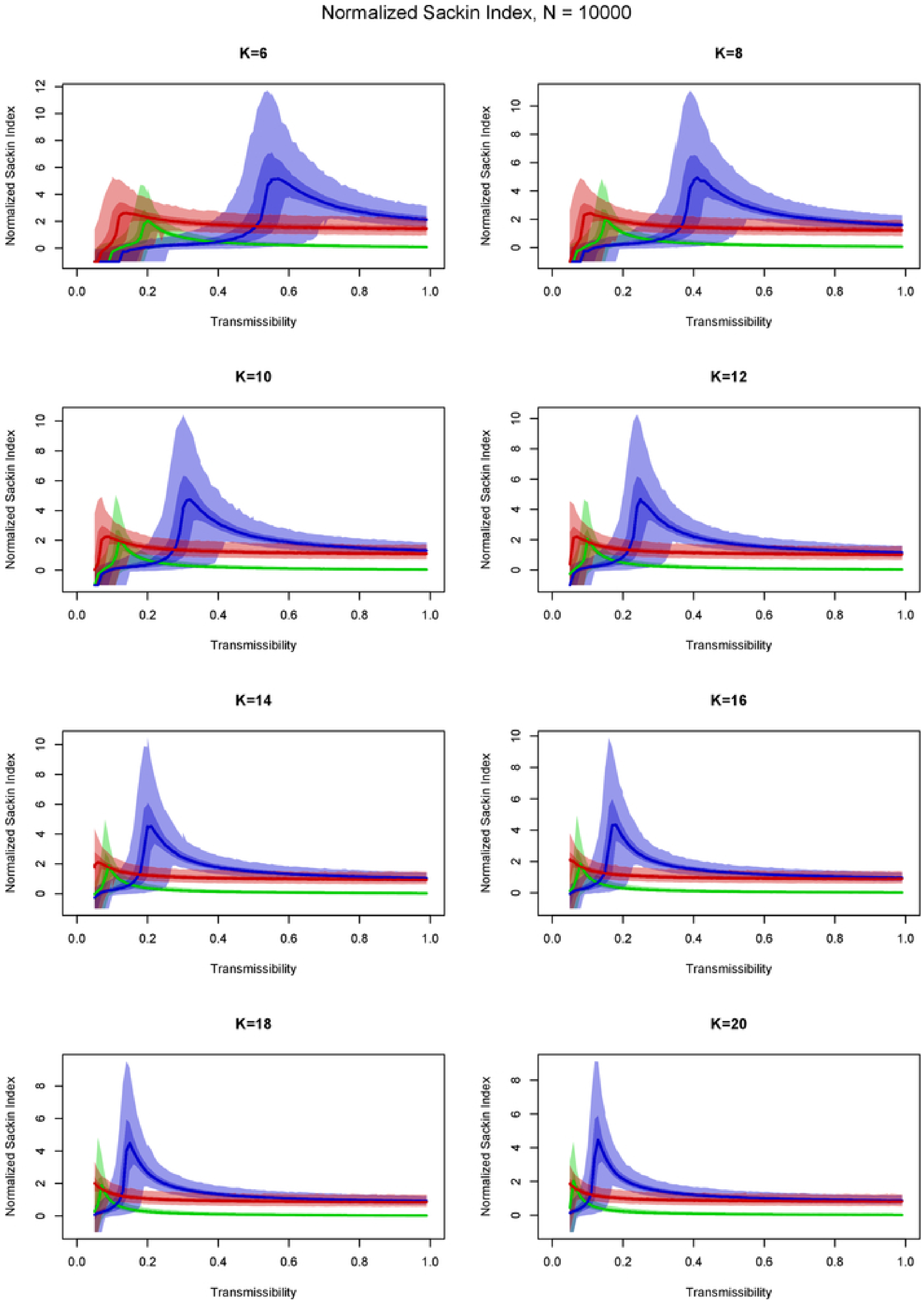
Normalized Sackin Index, N = 10000
K=6
K=8
K=10
K=12
K=14
K=16
K=18
K=20
Normalized Sackin Index
Transmissibility
0.0
0.2
0.4
0.6
0.8
1.0
0
2
4
6
8
10
12

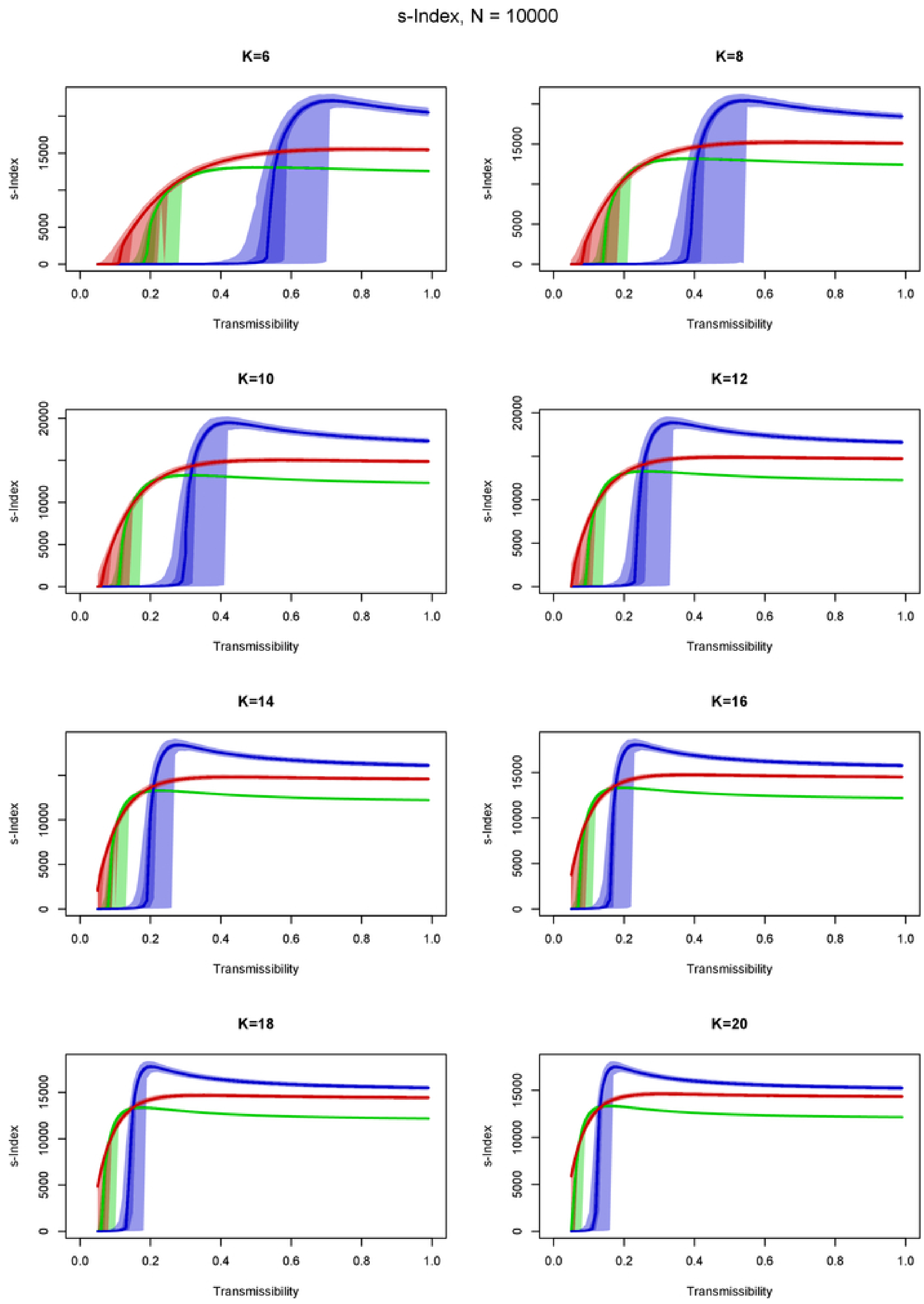
s-Index, N = 10000
K=6
K=8
K=10
K=12
K=14
K=16
K=18
K=20
s-Index
Transmissibility
0.0
0.2
0.4
0.6
0.8
1.0
0
5000
10000
15000
20000
